# Comprehensive review of dibenzocyclooctadiene lignans from the *Schisandra* genus: anticancer potential, mechanistic insights and future prospects in oncology

**DOI:** 10.1186/s13020-024-00879-0

**Published:** 2024-01-24

**Authors:** Karolina Jafernik, Sara Motyka, Daniela Calina, Javad Sharifi-Rad, Agnieszka Szopa

**Affiliations:** 1 Chair and Department of Pharmaceutical Botany, Jagiellonian University, Medical College, Medyczna 9 St., 30-688 Kraków, Poland; 2 Doctoral School of Medical and Health Sciences, Medical College, Jagiellonian University, Łazarza 16 St., 31-530 Kraków, Poland; 3 Department of Clinical Pharmacy, University of Medicine and Pharmacy of Craiova, 200349 Craiova, Romania; 4 Facultad de Medicina, Universidad del Azuay, Cuenca, Ecuador

**Keywords:** *Schisandra* lignans, Bicyclol, Anticancer activity, Dibenzocyclootadiene lignans, Apoptosis, Molecular mechanisms

## Abstract

Cancer remains one of the leading causes of mortality worldwide. The search for novel and effective anticancer agents has been a significant area of research. Dibenzocyclooctadiene lignans (DBCLS), derived from the *Schisandra* genus plants like: *S. chinensis, S. sphenanthera, S. henryi, S. rubriflora, S. grandiflora, S. propinqua,* and *S. glabra*, have been traditionally used in various medicinal systems and are known for their myriad health benefits, including anticancer properties. This comprehensive review aimed to collate and critically analyse the recent literature on the anticancer properties of DBCLS, focusing on their mechanistic approaches against different cancer types. An exhaustive literature search was performed using databases like PubMed/MedLine, Scopus, Web of Science, Embase, TRIP database and Google Scholar from 1980 to 2023. Peer-reviewed articles that elucidated the mechanistic approach of these lignans on cancer cell lines, in vivo models and preliminary clinical studies were included. Studies were assessed for their experimental designs, cancer types studied, and the mechanistic insights provided. The studies demonstrate that the anticancer effects of DBCLS compounds are primarily driven by their ability to trigger apoptosis, arrest the cell cycle, induce oxidative stress, modulate autophagy, and disrupt essential signaling pathways, notably MAPK, PI3K/Akt, and NF-κB. Additionally, these lignans have been shown to amplify the impact of traditional chemotherapy treatments, suggesting their potential role as supportive adjuncts in cancer therapy. Notably, several studies also emphasise their capacity to target cancer stem cells and mitigate multi-drug resistance specifically. DBCLS from the *Schisandra* genus have showcased significant potential as anticancer agents. Their multi-targeted mechanistic approach makes them promising candidates for further research, potentially leading to developing of new therapeutic strategies in cancer management.

**Supplementary Information:**

The online version contains supplementary material available at 10.1186/s13020-024-00879-0.

## Introduction

Compounds of plant origin are increasingly sought after for the prevention and treatment of cancer due to their availability and safety compared to synthetic chemotherapeutics [1]. The rising incidence of cancer, often classified as a civilization disease, is more prevalent in both developed and developing countries [2, 3]. Dibenzocyclooctadiene lignans (DBCLS), a significant group of compounds found in the genus *Schisandra* (family Schisandraceae), are known for their unique chemical structure and varied biological activities [4]. This group includes 40 identified secondary metabolites such as gomisin A, schisandrin B, schisandrin C, and ɤ-schisandrin, which exhibit hepatoprotective and hepatoregenerative properties [5–7]. Moreover, antiviral activity has been confirmed for deoxyschisandrin and ɤ- schisandrin [8, 9], antioxidant activity for schisandrin and schisandrin B [10, 11], and anti-inflammatory activity for schisandrin C and gomisin A [12, 13]. Recent research efforts have shifted towards exploring the anti-tumor potential of DBCLS and their role in inhibiting cancer cell growth. This article focuses on compounds such as gomisin A, gomisin G, schisandrin B, schisanhenol, gomisin L1, gomisin J, and schisantherin A, which have shown significant implications in cancer therapy. These compounds have been isolated and quantified from various *Schisandra* species, including *S. chinensis, S. sphenanthera, S. henryi, S. rubriflora, S. grandiflora, S. propinqua*, and *S. glabra*. The goal of this work is to gather and discuss the latest scientific reports on the anti-cancer potential of these isolated compounds and extracts, presenting a comprehensive overview of the current research in this field. In addition to detailing the general characteristics of selected *Schisandra* species and their biological significance, the review presents an in-depth look at the characteristics of selected DBCLS. A critical assessment of the reviewed results is included, complemented by descriptions of the latest biotechnological research. These advancements demonstrate the feasibility of producing these valuable compounds through plant cell tissue and organ cultures, underscoring the innovative approaches in plant biotechnology for obtaining DBCLS. This review thus contributes to expanding our understanding of this specific, rare, and still underexplored group of compounds, highlighting their biological activities and the innovative solutions for their production.

## Review methodology

This study aims to systematically review the literature on anticancer activity and the underlying mechanism of action of dibenzocyclooctadiene lignans derived from the *Schisandra* genus in the next databases: PubMed/MedLine, Scopus, Web of Science, Embase, TRIP database and Google Scholar using the following keyterms: “dibenzocyclooctadiene lignans”, “*Schisandra*”, “anticancer”, “tumor”, “neoplastic”, “mechanism”, “pathway”, “cytotoxicity” and “apoptosis”. We included the published articles from January 1980 to May 2023; original research articles in vitro*, *in vivo; studies with DBCLS on cancer cells, animal models with cancer, or human subjects related to cancer; use of dibenzocyclooctadiene lignans sourced from the *Schisandra* genus for cancer treatment or study; anticancer effects, cellular and molecular mechanisms, pathways of action, or any related insights regarding the anticancer properties of the mentioned lignans. Exclusion criteria: conference abstracts, case studies; non-English articles, unless they have an English abstract that provides sufficient detail; studies with insufficient data or where full text is not accessible; studies that do not directly assess the anticancer properties or the related mechanism of action of the dibenzocyclooctadiene lignans. For each selected article, the following data has been extracted: year of publication, country of origin; type of study (in vitro*, *in vivo*,* clinical), sample size, control and experimental groups; the specific cancer type studied (e.g., breast cancer, lung cancer, etc.); dosage, frequency, route of administration, and any other relevant specifics regarding the use of dibenzocyclooctadiene lignans; findings related to anticancer effects, mechanisms, pathways, cytotoxicity, apoptosis, and other related results. The most representative data have been summarised in tables and figures.

### Plant sources of DBCLS and their phytochemical profile, traditional uses and current medical applications

In contemporary therapy the use of isolated DBCLS is limited. Most often used the extracts from plant material derived from raw materials obtained from the *Schisandra* plants, namely: *Schisandra chinensis, Schisandra sphenanthera*, *Schisandra henryi, Schisandra rubriflora, Schisandra propinqua, Schisandra grandiflora* and *Schisandra glabra.* Promising results of DBCLS production are connected with plant biotechnology methods of their obtaining by in vitro tissue cultures of species from the *Schisandra* genus (Additional file 1). These studies are characterized in the Additional file (Additional file 1). Below the short characteristics of these plants, their occurrence, raw materials, chemical composition and studies on biological activity with special attention to anticancer potential are provided.

### *Schisandra chinensis*

The most popular species from the *Schisandra* genus, while highly valued for its medicinal properties, is *Schisandra chinensis* Turcz. Baill. (Chinense magnolia vine) [14]. Natural habitats of *S. chinensis* are located in Northeast China, Japan and Korea, as well as in the eastern part of Russia, in Primorsk, on the Kuril Islands, and in the south part of the island of Sakhalin. This species is usually found on the periphery of mixed forests [14]. The species has long been used in traditional Chinese medicine (TCM), but also in Korea, Japan and Russia. The raw material used in medicine is the fruit of *S. chinensis*—*Schisandrae chinensis fructus* (Northern Magnolia vine Fruit), which has monographs in the Chinese, Korean, Japanese and European Pharmacopoeias [15–21]. *Schisandra chinensis* fruit monograph is also listed in the WHO (World Health Organization) monograph [22]. According to the CosIng database, raw materials from *S. chinensis* can be used in the cosmetics industry in as many as 30 forms [23]. EFSA (European Food Safety Authority) gave a positive opinion on *S. chinensis* as a food additive or directly for consumption—has been found to have a good effect on well-being and strengthen mental immunity [24]. EMA (European Medicines Agency) has published a document confirming that *S. chinensis* can be an adaptogen [24]. Also, the FDA (U.S. Food and Drug Administration) allows the marketing of S*. chinensis* fruits in medicines and food [25]. *S. chinensis* fruits have been long used in TCM under the name Bei-Wuweizi—a fruit with five flavours [26–28]. It was believed that the salty and sour taste is responsible for improving the function of the liver and male gonads, the tart and bitter taste affects the work of the heart and lungs, and the sweet taste is responsible for the proper functioning of the stomach. *S. chinensis* fruit traditionally has been used to treat male impotence, frequent urination, and gonorrhea. It has been used to treat gastrointestinal diseases such as chronic diarrhea [29]. It was beneficial for respiratory system failure—asthma, wheezing and shortness of breath, cough, excessive phlegm production and diseases related to the circulatory system. *S. chinensis* was used as an adaptogenic agent, but also in states of excessive sweating and insomnia. The *S. chinensis* fruit was also used in traditional Russian medicine, where it is described as a tonic, that reduces hunger, thirst and fatigue [29, 30]. *S. chinensis* is the best-studied species in terms of chemical composition and biological potential. Researchers are focused primarily on analyzing the chemical composition of *S. chinensis* fruit extracts. The largest group of compounds found in fruit extracts are DBCLS (so called “*Schisandra chinensis* lignans”). It is said that there are even 30 compounds from this group [22, 31]. Further research on these compounds confirmed new comopounds from the group of lignans: schisanchinins A–D [32] and a lignan containing the nicotinoyl group—nicotinoylgomisin [33] (Table 1). Other types of lignans have also been found in *S. chinensis* fruits: tetrahydrofuran lignans: schinlignins A and B and dibenzylbutane lignans: schineolignans A-C [34, 35] (Table 1). In addition to research on the isolation of compounds from lignan groups, researchers are also focused on finding other compounds that also show high biological activity. The phytochemical profile of *S. chinensis* fruit extracts confirmed the presence of compounds belonging to the group of triterpenoids: preschisanartanes—schisanartanins A and B and 3,4-seco-21,26-olido-artan acid triterpenoid-wuweizilactone [34, 36–38]. Another group of compounds discovered in fruits is bisnortriterpenoids—wuweizidilactone [34] (Table 1). *S. chinensis* fruit extracts also contain about 3% essential oil. The composition also includes bioelements (Co, Mg, Fe, Zn, Cr, Ni, Cu, Ca, Mg, Fe, Mn, B) [26, 39, 40] (Table 1). In addition to the chemical profile of the fruits, researchers are also focusing on analysing the chemical profile of *S. chinensis* leaf and stem extracts. It has been shown that extracts from the leaves and stems of *S. chinensis* are rich in compounds from the triterpenoid group: schinchinenlactones A–C and schinchinenins A–H [41]. Two schinesdilactone-type nortriterpenoids were also identified: isoschicagenin C, schicagenins A–C and schinesdilactones A, B. Moreover, 16,17-seco-preschisanartane nortriterpenoids: schisdilactones A–G [28, 42] (Table 1). DBCLS were also found in extracts from *S. chinensis* stems with leaves: schisandrin, gomisin A and J, pregomisin, angeloylgomisin H, Q [43] (Table 1). In leaf extracts there were also identified other compounds from DBCLS group, e.g. schisantherin, deoxyschisandrin, schisandrin B and gomisin G [44–48] (Table 1). Leaf extracts also contained compounds from the group of flavonoids: quercetin, quercetin, isoquercitrin, hyperoside, rutin, myricetin and kaempferol [49]; besides, there are also glycosides such as quercetin 3-o-β-l-rhamnopyranosyl (1 → 6)-β-d glucopyranoside and ( +)-isoscoparin (Table 1). Leaf extracts are also rich in phenolic acids, such as protocatechuic, salicylic, syringic, chlorogenic, *p*-coumaric,* p*-hydroxybenzoic acids [50], as well as ferulic and gentisic acids [49] and the precursor of one group was also marked with phenolic acids—cinnamic acid [51] (Table 1). *S. chinensis* fruit extract, in addition to research on anticancer activity, is recognized as a hepatoprotective agent. Researchers have been focusing on the mechanism of hepatoprotective activity of individual lignans. It has been shown that gomisin A increases microsomal activity, e.g. N-demethylase, cytochrome B5 and 450. It also increases hepatocyte division, endoplasmic reticulum and hepatic blood flow [22, 30]. Another lignan tested for protective activity was γ-schisandrin. The compound increased the level of mitochondrial glutathione concentration and increased the level of vitamin C in the serum of the tested animals. Schisandrin B has also been tested for liver protection. It has been shown to protect the organ from oxidative stress [52]. The research also focused on the complex of lignans: schisantherin A, gomisin A, deoxyschisandrin, schisandrin, schisandrin B and schisandrin C. Their effect on the liver, which was induced by acetaminophen, was tested. The results showed that all lignans are hepatoprotective. The mechanism of their action is probably related to the inhibition of acetaminophenone bioactivation by cytochrome [53]. It was also shown that the polysaccharide isolated from the fruit extract of *S. chinensis* has a significant effect on lowering the concentration of aspartate aminotransferase and alanine aminotransferase in the serum of a mouse model with ethanolic liver injury. The polysaccharide also lowered the level of triglycerides in the liver and improved hepatological changes in the liver. Moreover, superoxide dismutase was increased in serum, HepG2 cells and ethanol-induced liver tissues [54]. It has also been shown that lignans isolated from *S. chinensis* extract have strong anti-inflammatory activity. The mechanism of action is based on the reduction of nitric oxide activity and prostaglandin synthesis by stimulating the release of cyclooxygenase and inhibiting the expression of nitric oxide synthase [32]. It has been shown that DBCLS have an inhibitory activity on microsomal lipid peroxidation, reduce the level of hydrogen peroxide and reduce the level of microsomal NADPH oxidation in hepatocytes [55]. Significant research is being conducted towards the adaptogenic and ergogenic effects of *S. chinensis* fruit extract. It has been shown that DBCLS present in the extract have a protective effect on nerve cells, preventing their death and preventing cognitive impairment in diseases related to the nervous system. The polysaccharides present also increase the accumulation of neurotransmitters in the central nervous system [56, 57]. The neuroprotective effect of isolated compounds from the root extract of *S. chinensis* has also been demonstrated. A new phenylpropanoid schineolignin D was isolated, a new sesquiterpene (-)-(7S,10S)-3,11,12,13-tetrahydroxycalamenene, a new quinic acid 4-(E)-o-coumaroylquinic ethyl ester. The compounds were tested for neuroprotective activity on H_2_O_2_-induced PC12 cell lines. Schineolignin D and quinic acid 4-(E)-o-coumaroylquinic ethyl ester have been shown to have significant neuroprotective effects [58]. *S. chinensis* fruit extracts have also been tested for antidepressant effect. Tests were carried out with the whole extract, isolated lignans, polysaccharides and essential oil. The research was carried out on mice subjected to forced swimming and tail suspension tests. It was shown that the whole extract and isolated lignans significantly shortened the immobility time of the animals. The mechanism of action in both cases was based on reducing the level of pro-inflammatory cytokines in the peripheral and central nervous system and inhibiting the go TLR4/NF-κB/IKKα signalling pathway [59]. Schisandrin A was isolated from the fruit extract of *S. chinensis*, which showed a significant therapeutic effect in diabetic nephropathy. Schisandrin A has been shown to reduce oxidative stress and inflammation in a mouse model of diabetic nephropathy, moreover, schisandrin A reduced ferroptosis caused by high glucose levels and pyroptosis caused by reactive oxygen species [60].

**Table 1:** Phytochemical profiles of individual species of the *Schisandra* genus

Species	Part of plant	Group of compounds	Chemical compounds	References
*Schisandra chinensis*	Fruits	DBCLS	Schisandrin, schisandrins B, C, γ-schisandrin, schisantherins A, B, schisanthenol, deoxyschisandrin, gomisin A, G, schisanchinins A–D, nicotinoylgomisin	[22, 31]
		Dibenzylbutane lignans	Schineolignans A–C	[34, 35]
		Tetrahydrofuran lignans	Schinlignin A, B	[34, 35]
		Triterpenoids	Schisanartanin A, B, schisanartanin A, B, 3,4-seco-21,26-olido-artan acid triterpenoid-wuweizilactone	[34, 36–38]
		Bisnortriterpenoids	Wuweizidilactone	[34]
		Bioelements	Co, Mg, Fe, Zn, Cr, Ni, Cu, Ca, Mg, Fe, Mn, B	[26, 39, 40]
	Leaves and stems	Triterpenoids	Schinchinenins A–H, schinchinenlactones A–C	[41]
		Nortriterpenoids	Schinesdilactone A. B, isoschicagenin C, schicagenins A–C, schisdilactones A–G	[28, 42]
		DBCLS	Schisandrin, gomisin A, J, pregomisin, angeloylgomisin H, Q	[43]
	Leaves	DBCLS	Deoxyschisandrin, schisandrin B, gomisin G, A, schisantherin, schisandrin	[44–48]
		Flavonoids	Isoquercitrin, hyperoside, rutin, myricetin, quercetin, quercetin and kaempferol glycosides: ( +)-isoscoparin, quercetin 3-o-β-l-rhamnopyranosyl (1 → 6)-β-d glucopyranoside	[49]
		Phenolic acids	Chlorogenic, *p*-coumaric, *p*-hydroxybenzoic, protocatechuic, salicylic, syringic, gentisic, ferulic, cinnamic acids	[49]
*Schisandra sphenanthera*	Fruits	DBCLS	Pregomisin, gomisin C, S, K3, J, U, epigomisin O, 6-o-benzoylgomisin, benzoylgomisin U, methylgomisin, tigloylgomisin P, angeloylgomisin P	[63]
		Derivatives of DBCLS	Schisfenins C–G, schisfenone	[65, 66]
		4-Aryltetralin lignan	Schisandrone	[65, 66]
		Aryltetralone lignan	Schisphentetralone A	[65, 66]
		3-Dimethyl-1,4-diarylbutane lignans	d, l-Anwulignan, sphenanlignan	[65, 66]
		2,5-Diaryltetrahydrofuran lignans	Chicanin, d-epigalbacin, ganschisandrine	[65, 66]
		Triterpenoids	Schisandronic acid (ganvuweisic acid), anvuweisic acid, kadsuric acid, coccinic acid, schinalactones A–C, G, schisanol	[65, 66, 69]
		Nortriterpenoids	Sphenalactones A–D, sphenadilactone C, sphenazine	[70, 117]
	Stems	DBCLS	Gomisin B, G, O, epigomisin O, schisantherin A, D, marlignan E, angeloylgomysin Q, schisfenlignans A–D	[67]
	Roots	Terahydrofuran lignans	Schiglaucin A, B, epoxyzuonin, thalaumidine, myristartenol A	[68]
*Schisandra henryi*	Leaves and stems	DBCLS	Gomisin G, schisantherin A, benzylgomisin Q, deoxyschisandrin, schisandrin	[78–80]
		Aryltetraline lignans	Wulignan A1, A2, epiwulignan A1, enshicine, epienshicine, dimethylwulignan A1	[78–80]
		Dibenzylbutane lignans	Henricin A, B, isoanwulignan	[78–80]
		Tetrahydrofuran lignan	Ganschisandrine	[80]
	Stems	Triterpenoids	Henrisquinines A–C, acids: isoschisandronic, kadsuric, anwuweizic, schisandronic, nigranic, 3-ethyl ester of nigranic acid, schisanlactone B	[81]
		Nortriterpenoids	Henridilactones A-D, schiprolactone A	[82]
	Fruits	DBCLS	Schisantherin B, schisanhenol, schisanhenrin	[83]
		Terpenoids acids	Kadsuric acid, schisanhenric acid	[83]
	Leaves	DBCLS	Schisandrin, gomisin G, schisantherin A, B, deoxyschisandrin, schisandrin C,	[77]
		Phenolic acids	Gallic, neochlorogenic, kaftaric, caffeic acids	[77]
		Flavonoids	Hyperoside, rutin, trifolin, quercitrin, quercitin and kaempferol	[77]
*Schisandra rubriflora*	Fruits	DBCLS	Angeloylgomisin P, Q, benzoylgomisin Q, deoxyschisandrin, epiwulignan A1, isogomisin O, rubrilignans A, B, rubrischisantherin	[88]
		Phenolic acids	Chlorogenic acid, cryptochlorogenic acid	[89]
		Flavonoids	Hyperoside, rutin, isoquercitrin, guaijaverin, trifolin, quercetin, kaempferol, isorhamnetin	[89]
	Leaves	Dibenzylbutane lignans	Mesomono-methyl-dihydroguaretic acid, mesodihydroguaretic acid, 4,4′-(2R,3S)-2,3-dimethylbutane-1,4-diyl)bis(1,2-dimethoxybenzene)	[86]
		Arylnaphthalene lignans	(8R,7′R,8R)-5-Hydroxy-4,3′,4′-trimethoxy-2,7′-cyclolignan	[86]
		Flavonoids	Hyperoside, rutin, isoquercetin, guaijaverin, trifolin, quercetin, kaempferol, isorhamnetin	[88]
	Stems	DBCLS	Angeloylgomisin Q and P, schisandrin, schisantherin A, gomisin D, epigomisin O, tiglomisin P, benzoylgomisin Q, machilin D, rubrisandrin A, B, schirubrins A–D	[86]
		Megastiman glycosides	Megastigmano-3-α-4β-9ξ-triol-3-o-β-d-glucopyranoside and 7-megastigmano-3-ol-9-one-3-o-α-l-arabifuranosyl-(1 → 6)–β–d-glucopyranoside	[87]
		Phenolic acids	Syringic, neochlorogenic, chlorogenic, cryptochlorogenic acids	[86, 88]
		Flavonoids	Naringin, didimine, mazopsin 6-o-glucopyranoside, hyperoside, rutine, isoquercetin, guaijaverin, trifolin, quercetin, kaempferol, isorhamnetin	[86, 88]
		Bisnortriterpenoids	Rubriflordilactones A, B	[86, 88]
	Stems and leaves	Nortriterpenoids	Rubriflorins A–J	[86, 88]
*Schisandra grandiflora*	Fruits	DBCLS	Gomisins A, B, D, K3, M1 and N, deoxyschisandrin, schisandrin, (–) gomisin K1, epigomisin O, tiglomisin P, benzoylgomisin O, schisandrin C	[97–99]
		Tetrahydrofuran lignans	Chicanine	[97–99]
		Derivatives of tetrahydrofuran lignans	Veraguensin	[97–99]
		Dibenzylbutane lignans	Macelignan	[97–99]
		Diaryldimethylbutane lignans	Saurulignan B	[97–99]
		Tetralin lignans	Arisantetralone C. D	[97–99]
		Triterpenoids	Schisandronic acid, schizandrolic acid	[97–99]
		Sesquiterpenoids	Viddaranal A–C, isokuparenal, schisanspheninal A, vidarol peroxide	[96]
	Leaves and stems	Nortriterpenoids	Schigrandilactones A–C, lancifodilactones C, D, 11K, L, N, 12 microrandilactones A,2, henridilactones A, B	[100]
	Stems	Triterpenoids	Granditriol, 2α,3β,23-trihydroxyurs-12,20(30)-dien-28-oic acid β-d-glucopyranosyl ester, acetylursolic acid, ursolic acid, 2α,3α-dihydroxyurs-12-ene-28-oic acid, corosolic acid, asiatic acid, 2α,3α,19α-trihydroxyurs-12-ene-28-oic acid, 2α,3α,23-trihydroxyurs-12-ene -28-oic acid, 23-hydroxyursolic acid, maslinic acid, 2α,3β,23-trihydroxyolean-12-en-28-oic acid β-d-glucopyranosyl ester, lupeol, betulinic acid	[101]
*Schisandra propinqua*	Stems	DBCLS	Interioterin A, benzoylgomisin O, gomisin G, O schisantherin, heteroclithin A, tigloylgomisin P, angeloylgomisin O, angeloylisogomisin O, cadsulignan L(4), ( ±) 5,8-epoxy-6,7-dimethyl-2′,3′,2″,3″-dimethylenedioxy-4′,1″-dimethyl-1,2:3,4-dibenzo-1,3-cyclooctadiene, wuweizisu C, angeloyl-( +)-gomisin K, methylisogomisin O, isogomisin O, angeloylisogomisin O, angeloygomisin O, benzoylgomisin O, epigomisin O, propinvanins A–D	[102–106]
		Neolignans	4,4-Di(4-hydroxy-3-methoxyphenyl)-2,3-dimethylbutanol	[132]
		Derivatives of lignans	Galgravin, veraguensin, octadecanoic acid 2,3-dihydroxypropyl ester, 2-hexadecanoic acid 3-dihydroxypropyl ester, tetracosanoic acid 2,3-dihydroxypropyl ester	[102]
		Triterpenoids acids	Schisandrolic, isoschisandrolic, nigranic, manvuweic, schisandronic acids	[118]
		Nortiterpenoids	Propindilactones E-J	[110]
	Stems and leaves	Triterpenoids	Propindilactone T, U, changnanic acid 3-methyl ester, schipropine acid	[136]
	Leaves	Nitrophenol glycosides	6′-o-Alpha-l-arabinofuranosylthalictoside, 6′-o-beta-d-apiofuranosylthalictoside, talictoside, icarizide D2, prinsepiol, ( +)-1-hydroxypinoresinol, ( +)-medioresinol	[112]

### *Schisandra sphenanthera*

The second most popular species of the *Schisandra* genus is *Schisandra sphenenthera* Rehd. et Wils. This vine plant grows mainly in the Qinling Mountains that separate North China from South China along the Huai River, as well as in Taiwan and Vietnam [61]. *S. sphnenatnhera*, like *S. chinensis* is widely used in TCM. Often the two species were confused with each other due to the small differences in morphological appearance. The raw material of *S. sphneanthera* with a therapeutic effect is also fruit—*Schisandrae sphenantherae fructus* (Southern Magnolia vine Fruit) having monographs in the Chinese Pharmacopoeia [20]. In folk medicine, *Schisandrae sphenanthera* is called Nan-Wuweizi. Used to reduce nervous tension, strengthen the kidneys, as an expectorant and strengthen „Qi” (the vital energy in TCM) [62].

*Schisandrae sphenanthera* fruit extracts according to traditional indication are also used in hepatitis, osteoporosis and insomnia [63]. *S. sphenanthera* is also the object of a large number of studies on the phytochemical profile and bioactivity. In fruit extracts, the dominant group of secondary metabolites are DBCLS, including: gomisin C, S, K3, J and U, pregomisin, benzoylgomisin U, methylgomisin, tigloylgomisin P, epigomisin O, 6-O-benzoylgomisin and angeloylgomisin P [63] (Table 1).

The presence of derivatives of DBCLS, i.e. C18-dibenzocyclooctadiene lignans—schisphenins C–G and 6,7-seco-dibenzocyclooctadiene lignan—schisphfenone [64], was also confirmed in the extracts from *S. sphenanthera* fruits. Studies have also shown the presence of other groups of lignans: 4-aryltetralin lignans (schisandrone), aryltetralone lignans: schisphentetralone A, 2,3-dimethyl-1,4-diarylbutane lignans: sphenanlignan, D, l-anwulignan; and three 2,5-diaryltetrahydrofuran lignans: chicanin, d-epigalbacin and ganschisandrin [65, 66] (Table 1). Research was also focused on the phytochemical profile of *S. sphenanthera* stems. The presence of, in addition to the previously known, epigomisin O, gomisin B, G, O, marlignan E schisantherin A, D, and angeloylgomysin Q, as well as the new DBCLS—schisphenlignans A–D were confirmed [67] (Table 1). Research on *S. sphenanthera* root extracts indicated the presence of schiglaucins A, B, epoxyzuonin, myristartenol A, thalaumidine and eight new, previously unknown tetrahydrofuran lignans [68] (Table 1). In addition to lignans, the phytochemical profile of *S. sphenanthera* fruit extracts is also characterized by the presence of compounds from the following groups: triterpenoids: schisandronic acid (ganvuweisic acid), anvuweisic acid, kadsuric acid, coccinic acid, schinalactones A-C and G, and schisanol [65, 66, 69] and nortriterpenoid compounds: sphenadilactone C, sphenazine A and sphenalactones A–D [67, 70] (Table 1). *S. sphenanthera* extracts show strong hepatoprotective effect, which is related to antiviral activity against chemical hepatitis and against toxins that attack hepatocytes [7, 71]. Studies have shown that DBCLS (schisantherin A, B, C and D), which has been isolated from fruit extract, lower the level of glutamate pyruvate transaminase in the serum of patients with chronic viral hepatitis [72]. It has also been shown that extracts from *S. sphenanthera* may have an antioxidant potential. The study isolated the hydrophilic polysaccharide SSPP11. It was found that it has inhibitory activity on lipid peroxidation and protective activity on oxidative degradation of proteins [73, 74]. Nowadays, extract from fruits is also used in the cosmetics industry. The CosIng database states that the extract can be used as an antioxidant, skin protecting and condictioning and anti-sebum [23].

### *Schisandra henryi*

The lesser known and naturally widespread only in the Yunnan Province of China *Schisandra* genus is *Schisandra henryi* C.B. Clarke. In TCM, it was used as a substitute for the fruit of *S. chinensis* [14, 75]. Despite its high therapeutic potential, it does not have a monograph in any pharmacopoeia. In folk medicine, the stems of *S. henryi* (yi geng wu wei zi) have been used to support blood circulation, and heal fractures and irregular menstrual cycles [14]. In the available literature, very few studies focus on the phytochemical profile of *S. henryi*. The works elaborate the analysis of the chemical composition of extracts from leaves and shoots. The main group of compounds found in *S. henryi* are lignans. The presence of lignans from the DBCLS, as well as aryltetraline and dibenzylbutane lignans found in the extracts, were confirmed [75–77] (Table 1). The folowing lignans were identified in the leaves and stems; from DBCLS: gomisin G, schisantherin A, benzoylgomisin Q, deoxyschisandrin, schisandrin; from the aryltetraline group: enshicine, epienshicine, wulignan A1, A2, epiwulignan A1 and dimethylwulignan A1 and from the dibenzylbutane group: henricin A and B and isoanwulignan [78–80] (Table 1). In the shoots themselves, the presence of ganschisandrine—tetrahydrofuran lignan was also shown [80] (Table 1). In addition to lignans, the presence of triterpenoid compounds was found in the shoots: henrisquinine A, B and C and the acids: isoschisandronic, kadsuric and anwuweizic, as well as the 3-ethyl ester of nigranic acid, nigranic and schisandronic acids, schisanlactone B [81] as well as nortriprenoid compounds: henridilactones A-D and schiprolactone A [82] (Table 1). In the extract from fruits of *S. henryi*, compounds from the group of lignans were found: schisantherin B, schisanhenol, schisanhenrin and terpenoids: kadsuric acid, schisanhenric acid [83] (Table 1). An analysis of the chemical composition of *S. henryi* leaves was also carried out. The tested leaf extracts contained DBCLS: schisantherin A and B, gomisin G, deoxyschisandrin, schisandrin and schisandrin C, phenolic acids: gallic, neochlorogenic, kaftaric and caffeic; and flavonoids: quercitrin, quercitin, hyperoside, rutin, trifolin and kaempferol [77] (Table 1). There is also little research on the biological profile of *S. henryi* extracts. Researchers focused on examining the chemical composition of *S. henryi* and the biological activity of individual compounds. They isolated from the seeds of *S. henryi* two triterpenoid acids—kadsuric and schisanhenric acids, and thirteen lignans—wulignan A1 and A2, schisantherin A, schisanhenol, deoxschisandrin, schisantherin B, epienshicin methyl ether, epischisandrone, epiwulignan A1, schisandrone, enshicine, henricine and enshizhisu. It was shown that epiwulignan A1, wulignan A1, A2 and epischisandrone showed inhibitory activity against P-388 lymphoma cell lines [79]. Another study was based on the isolation of four compounds from dried stems of *S. henryi*—isowulignan, gomisin G, schisantherin A and benzoylgomisin Q and then testing the biological activity of these compounds on DNA strand cleavage and cytototoxic activity on leukemia and HeLa cell lines in vitro. It was shown that gomisin G in the presence of Cu^2+^ showed strong DNA cleavage activity at a concentration of 50 μg/mL, with over 50% relaxation of the supercoiled DNA. The other compounds showed no activity. In in vitro tests on cell lines, gomisin G was found to have the greatest cytotoxic effect (IC50 = 5.51 μg/mL) on leukemia and HeLa cell lines. Benzoylgomisin Q and schisantherin A showed a moderate cytotoxic effect on leukemia cells (IC50 = 55.1 and 61.2 μg/mL). As for cytotoxicity towards HeLa cell lines, benzoylgomisin Q showed a moderate effect (IC50 = 61.2 μg/mL, in contrast to schisantherin A, which was inactive [84]. Other studies focus on the antioxidant and anti-inflammatory activity of *S. henryi* extracts from microshoot cultures in vitro and in leaves. The total content of polyphenols was also determined. Antioxidant tests were carried out using the CUPRAC, FRAP and DPPH methods, anti-inflammatory activity was determined by the method of inhibiting the activity of enzymes: 15-LOX, COX-1, COX-2 and sPLA_2_. The total content of polyphenols was determined by the Folin-Ciocalteu spectrophotometric method. The obtained results showed that the total content of polyphenols in extracts from microshoot cultures was 1146 nmol/Gal/mg DW, in leaf extracts—277 nmol/Gal/mg DW. Antioxidant activity for extracts from microshoot cultures and leaves was respectively for the CUPRAC method: 259 and 67 TE nmol/mg DW, for FRAP: 135 and 24 TE nmol/mg DW, and for the DPPH method: 176 and 53 TE nmol/mg DW. The anti-inflammatory effect for the extracts from the microshoot and leaf cultures was respectively for sPLA_2_: 17% and 19% inhibition, for 15-LOX: 27% and 26%, for COX-1: 76% and 70%, for COX-2: 66% and 33% inhibition. The results suggest that the extracts from the microshoot cultures of *S. henryi* have a much greatest biological potential, which is associated with a greater accumulation of polyphenols. The researchers isolated 12 compounds from the leaves and stems of *S. henryi*, including eleven—henridilactones E-O—for the first time. Next, the obtained compounds’ biological potential was tested regarding neuroprotective effect by inducing apoptosis by corticosterone in PC12 cells. It was shown that four compounds had the greatest neuroprotective effect: henridilactones E, H, N and O—they effectively reduced cell apoptosis. In addition, henridilactone O increased the number of neurites [85].

### *Schisandra rubriflora*

*Schisandra rubriflora* Rehd. et Wils. is an endemic species that is naturally found only in the western province of Sichuan in China. *S. rubriflora* is most closely related to *S. grandiflora*—taxonomic differences are only in the color of the flowers. The flowers of *S. rubriflora* are red, those of *S. grandiflora* are cream [14]. *S. rubriflora* is cultivated in Asia, but outside the continent, cultivation of this species on a larger scale is very rare [14]. The *S. rubriflora* species does not have a monograph in any pharmacopoeia, nor is it listed in the WHO and is not used in the cosmetics industry [86]. In TCM, the fruit of *S. rubriflora* was used as a sedative and tonic. Probably the fruit was also used in ailments related to the liver, and digestive system and as an adaptogenic agent. The fruits of S. *rubriflora* are also considered a local delicacy to this day [86, 87]. The phytochemical profile of *S. rubriflora* is very interesting. From the available literature, it is known that the main metabolites present in the fruits of this species are lignans, primarily DBCLS, such as: angeloylgomisin P and Q, benzoylgomisin Q, deoxyschisandrin, epiwulignan A1, or isogomisin O [88] (Table 1). In addition, the fruits contain DBCLS group, characteristic only for this species: rubrilignans A and B, and rubrischisantherin [86] (Table 1). *S. rubirflora* fruit extracts were also tested for the content of polyphenolic compounds—phenolic acids and flavonoids. Chlorogenic and cryptochlorogenic acids as well as flavonoids: hyperoside, rutin, isoquercitrin, trifolin, guavaverin, kaempferol, quercetin and isorhamnetin, were confirmed [89] (Table 1). The presence of compounds such as mesomono-methyl-dihydroguaretic acid, mesodihydroguaretic acid and 4,4′-(2R,3S)-2,3-dimethylbutane-1,4-diyl)bis(1,2-dimethoxybenzene) was confirmed in the leaves of *S. rubriflora* belonging to the group of dibenzylbutane lignans. Also identified was (8R,7′R,8R)-5-hydroxy-4,3′,4′-trimethoxy-2,7′-cyclolignane, which is classified as arylnaphthalene lignans [86] (Table 1). In leaf extracts, polyphenolic compounds from the group of flavonoids were also identified: hyperoside, trifolin, rutoside, isoquercetin, guavaverin, quercetin, isorhamnetin and kaempferol [88] (Table 1). *S. rubriflora* microshoots were also subjected to phytochemical analysis. Among others, the following were identified: angeloylgomisin Q and P, schisandrin, schisanterin A, gomisin D, epigomisin O, tigloylmisin P, benzoylgomisin Q, machilin D and specific lignans: rubrisandrins A, B and schirubrins A–D [86] (Table 1). Megastiman glycosides—megastigmano-3-α-4β-9ξ-triol-3-o-β-d-glucopyranoside and 7-megastigmano-3-ol-9-one-3-o-α-l-arabifuranosyl-(1 → 6)-β-d-glucopyranoside [87] (Table 1). In addition, in the microshoots of *S. rubriflora*, phenolic acids such as syringic, neochlorogenic, chlorogenic and cryptochlorogenic acids were identified, as well as flavonoids, including: naringin, didimine or mazopsin 6-o-glucopyranoside and five flavonoid glycosides: hyperoside, rutoside, isoquercetin, trifolin and guavaverin, and three aglycones: and isorhamnetin, quercetin and kaempferol [86, 88] (Table 1). *S. rubriflora* is characterized by the presence of specific nortriterpenoids that have been identified in shoots and leaves, these are rubriflorins A–J. In the shoots of *S. rubriflora*, compounds belonging to bisnortriterpenoids were also identified: rubriflordilactone A and B (Table 1) [86, 88]. A detailed phytochemical profile of compounds found in *S. rubriflora* is presented in Table 1 [88, 89]. In addition to the anticancer activity of the lignans found in *S. rubriflora*, these compounds are being investigated for other biological activities. It has been shown that the activity inhibiting the replication of the HIV virus is exhibited by, among others: gomisin M1, gomisin J, gomisin M2, rubrisandrins A and B, rubriflordilactone B, rubriflorins A–J, schirubridillactones A–F and schisanhenol. These compounds are responsible, inter alia, for inhibiting HIV-1 replication in lymphocytes. The activity is mainly due to the presence of hydroxyl substituents in chemical structures [90–94]. It was also found that extracts from leaves, fruits and microshoots of *S. rubiriflora* have an effective inhibitory effect on pro-inflammatory enzymes: cyclooxygenases COX-1 and COX-2, 15-LOX andsPLA_2_ [88]. Studies have also been carried out to confirm the antioxidant power of *S. rubriflora* extracts. The highest antioxidant potential was shown by the leaf extract using the FRAP and CUPRAC, QENCHER-CUPRAC and DPPH assays [89].

### *Schisandra grandiflora*

*Schisandra grandiflora* Hook. f. & Thoms., is rare and a little-known species in Europe. It is widespread in India (Himachal Pradesh, Uttar Pradesh, Sikkim, northwestern Bengal), Nepal, Bhutan and southern Tibet. The natural occurrence of *S. grandiflora* is the Qinling Mountains [14]. It is a type of dioecious climber growing on slopes of the mountain, most often in deciduous forests [14]. *S. grandiflora* is one of the most attractive species of the *Schisandra* genus due to its commercial value—it is grown as an ornamental plant with large, strongly scented flowers and fruits. The species does not have its monograph in any of the Pharmacopoeias, it is not used in cosmetic preparations [14, 95, 96]. In TCM, the fruits of *S. grandiflora* were used to treat various ailments, most often related to liver diseases—research confirms the presence of DBCLS lignans, which have a broad hepatoprotective effect. The community also used the edible fruit of *S. grandiflora* as a delicacy and used it in food for its clove flavor [14, 95, 96]. As for the species S*. grandiflora*, as with *S. henryi*, not too many studies on chemical composition analysis. Most studies concern the analysis of the chemical composition of *S. grandiflora* fruit extracts. As in the case of other species of the genus *Schisandra,* the largest group of compounds are DBCLS. The presence of gomisin K3, deoxyschisandrin, schisandrin C, schisandrin, (–) gomisin K1, epigomisin O, tigloylgomisin P, benzoylisogomisin O, gomisin B, gomisin N, gomisin M1, gomisin A and D was confirmed in the fruit. There are also other groups of lignans from the fruits of *S. grandiflora*: tetrahydrofuran—chicanine, and its derivatives—veraguensin, dibenzylbutane—macelignan, diaryldimethylbutane—saurulignan B, and tetralin—arisantetralone C and D. In addition, the presence of of triterpenoids—schisanhol, schisandronic and schizandrolic acids [97–99] (Table 1). Sesquiterpene compounds—viddaranal A, viddaranal B, viddaranal C, isokuparenal, schisanspheninal A and vidarol peroxide were also found in fruit extracts of *S. grandiflora* [96] (Table 1). The presence of nortriterpenoids was confirmed in leaf and steam extracts: schigrandilactones A–C, N, 12 microrandilactones A, 2, lancifodilactones C, D, 11 K, L and henridilactones A, B [100] (Table 1). The stems of *S. grandiflora* contain compounds from the group of triterpenoids: granditriol, 2α,3β,23-trihydroxyurs-12,20(30)-dien-28-oic acid β-d-glucopyranosyl ester, acetylursolic acid, ursolic acid, 2α,3α-dihydroxyurs-12-ene-28-oic acid, corosolic acid, asiatic acid, 2α,3α,19α-trihydroxyurs-12-ene-28-oic acid, 2α,3α,23-trihydroxyurs-12-ene -28-oic acid, 23-hydroxyursolic acid, maslinic acid, 2α,3β,23-trihydroxyolean-12-en-28-oic acid β-d-glucopyranosyl ester, lupeol, and betulinic acid [101] (Table 1). Also, the number of research on the biological potential of *S. grandiflora* extracts is small. Studies were conducted on the cytotoxic activity of nortriterpenoids: schigrandilactones A-C on two cancer cell lines: HepG2 and K562, and on the HIV-1 inhibitory activity in infected C8166 cells. It was shown that schigrandilactones A and B effectively inhibited the proliferation of cancer cells in the two tested cell lines, but they did not show inhibitory activity against HIV-1 [100]. Schigrandilactone C showed a moderate inhibitory effect on the development of both cancer cell lines; however, it showed a high inhibitory potential against HIV-1 [100]. Research was conducted in which gomisin B was isolated from S*. grandiflora* and a number of derivatives based on triazoles were created. It was found that the synthesised derivatives showed anticancer activity against SIHA cells and had a cytotoxic effect on them, better than the drug—doxorubicin [99]. The antioxidant activity of a chloroform extract from *S. grandiflora* fruit was also studied. Individual compounds were isolated and, based on biological tests, it was found that the highest activity of scavenging free radicals and inhibiting advanced glycation end compounds was shown by arisantetralone C and D, gomisin M1, arisantetralone C and D, saurulignan B, macelignan [97]. The triterpenoid compounds isolated from the stems of S*. grandiflora* were tested in terms of antifungal activity against the pathogens *Alternaria solani* and *A. alternata*. It was shown that asiatic acid and 2α,3α,19α-trihydroxyurs-12-en-28-oic acid had the strongest antifungal activity (MIC = 25 μg/mL). The bioactive compounds were also tested for antiproliferative activity against HepG2 cell lines in the same study. The highest antiproliferative activity against cancer cells was shown by the triterpenoid betulinic acid [101].

### *Schisandra propinqua*

*Schisandra propinqua* (Wall.) Baill. is widespread in southwestern China [14]. Although it is not as popular a species as *S. chinensis*, it is of great interest to researchers, which translates into quite a large number of studies on the chemical composition and biological activity of *S. propinqua* [102]. The raw material of *S. propinqua* used in TCM are rhizomes and stems administered orally or topically. They are used as anti-inflammatory and analgesic agents, but also in stomach problems, hepatitis, rheumatism. Despite many traditional indications, *S. propinqua* is not listed in pharmacopoeias, nor other official documents [102]. The phytochemical profile of *S. propinqua* focuses primarily on extracts from the above-ground parts of the plant. The chemical composition of this species is very interesting due to the large amount of compounds from the terpenoid group and their derivatives. A large group of compounds found in *S. propinqua* are also DBCLS. The phytochemical profile of the above-ground parts of *S. propinqua* is characterized by large amount of compounds from the terpenoid group and their derivatives, as well as DBCLS.The following lignans were confirmed in the stems: interiotherin A, benzoylogomisin O, gomisin G, schisantherin, heteroclitin A, tigloylgomisin O, angeloylgomisin O, angeloylisogomisin O, kadsulignan L, ( ±) 5,8-epoxy-6,7-dimethyl-2′,3′,2″,3″-dimethylenedioxy-4′,1″-dimethyl-1,2:3,4-dibenzo-1,3-cyclooctadiene, schisandrin C, angeloyl-( +)-gomisin K, methylisogomisin O, gomisin O, isogomisin O, epigomisin O, and characteristic for this species: propinvanin A–D. In stem extracts was aslo confirmed compound from the neolignan group: 4,4-di(4-hydroxy-3-methoxyphenyl)-2,3-dimethylbutanol [102–106]. Moreover, in stems the lignan derviatives were also detected: galgravin, veraguensin, octadecanoic acid 2,3-dihydroxypropyl ester, 2-hexadecanoic acid 3-dihydroxypropyl ester and tetracosanoic acid 2,3-dihydroxypropyl ester. In addition, cytotoxicity studies of these compounds were performed, which showed that the compounds have a significant inhibitory effect on vascular smooth muscle cell (VSMC) division in vitro [107]. From abundantly present tirterpenoids and their derivatives, the two tirterpenoid acids—schisandrolic acid and isoschisandrolic acid—were confirmed in the stems. Other tritperpenoid acids that have been isolated from *S. propinqua* stem extract are nigranic, manvuweic and schisandronic acid. In addition, tests for the cytotoxic activity of these compounds were carried out, which showed that nigranic acid and manvuweisic acid have a strong effect on cell lines [108]. Moreover, from the stems and leaves extracts, compounds with a triterpenoid structure were isolated and identified: changnanic acid 3-methyl ester, propindilactone U, propindilactone T and schipropine acid [109]. In the stems, were confirmed also compounds from the nortriterpenoids group of the structure of schisantrane [110]. These compounds were identified as propindilactones E–J and indicated as characteristic of the *S. propinqua* species [111]. Addtionally, a very specific group of compounds that has been isolated from *S. propinqua* leaf extract is nitrophenol glycosides: 6′-o-alpha-l-arabinofuranosylthalictoside, 6′-o-beta-d-apiofuranosylthalictoside, talictoside, icarizide D2, prinsepiol, ( +)-1-hydroxypinoresinol and ( +)-medioresinol [112]. Numerous unique compounds have been isolated from *S. propinqua*, future research will show what activities they may have. Now there is no such data.

### *Schisandra glabra*

*Schisandra glabra* (Brickell) Rehder is the only representative of the *Schisandra* genus naturally occurring in the North America [14, 113]. In America, other names for *S. glabra* are very common, such as: “Scarlet Woodbine”, “American Starvine”, “Bay Starvine”, “wild sarsaparilla”, “climbing magnolia” and “magnolia creeper” [114]. Moreover, the second latin name—*Schisandra coccinea*, is used interchangeably, which can often mislead the reader [113]. The range of *S. glabra* in the United States includes the southeastern states: South Carolina, North Carolina, Florida, Georgia, Kentucky, Alabama, Tennessee, Louisiana, Arkansas, Mississippi and the Hidalgo cloud forests, stretching along the Sierra Madre in Mexico.

*Schisandra glabra* is also widespread throughout East Asia. In the USA, *S. glabra* is defined as a 50% threatened or endangered species [115]. Despite the fact of high popularity of this plant as ornamental one, there is no information regarding the biological activities and phytochemical studies of *S. glabra* [113, 114, 116].

### Chemical structure of DBCLS

**Fig. 1:**
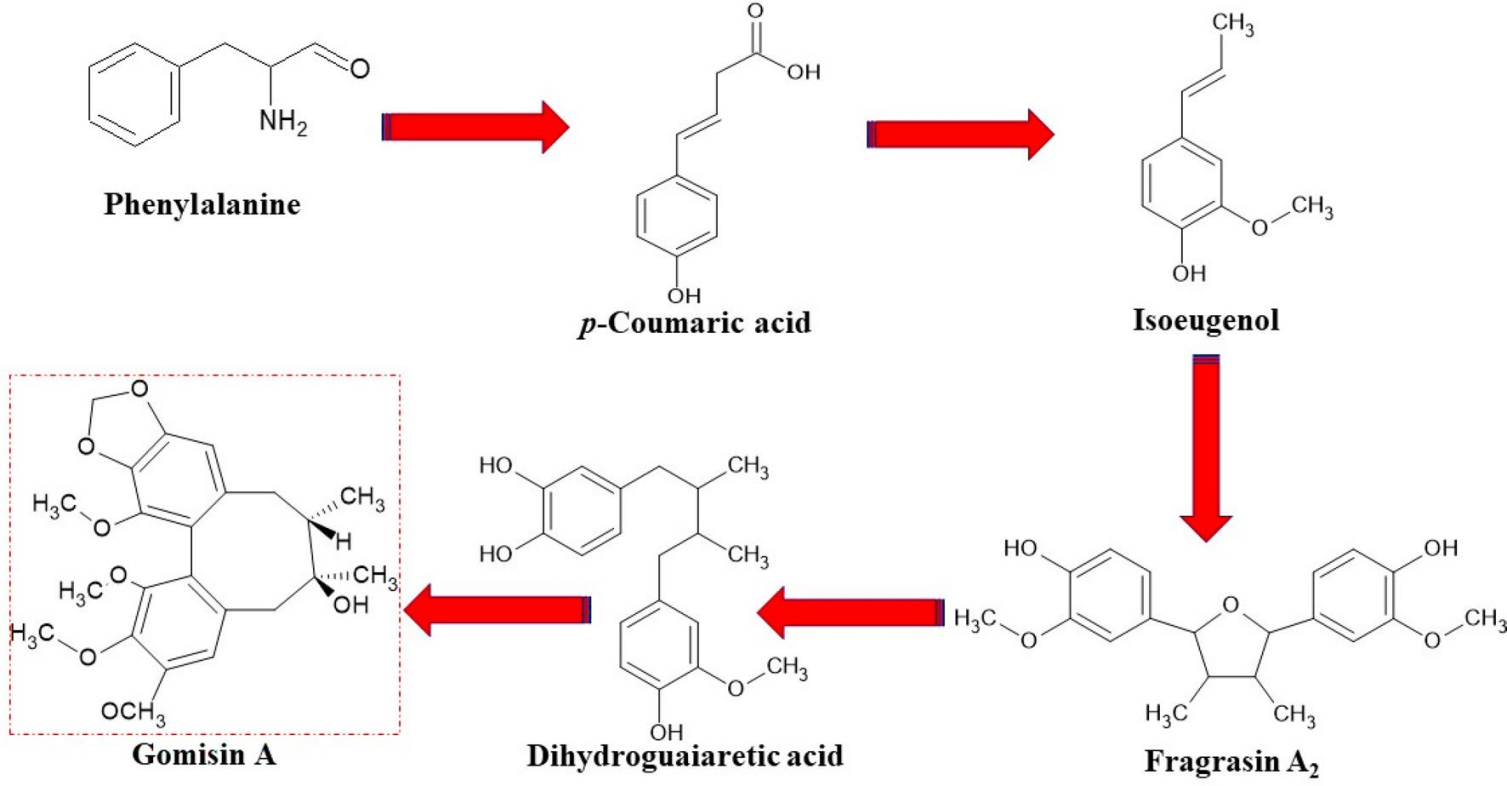
General biosynthetic pathway of DBCLS

**Fig. 2:**
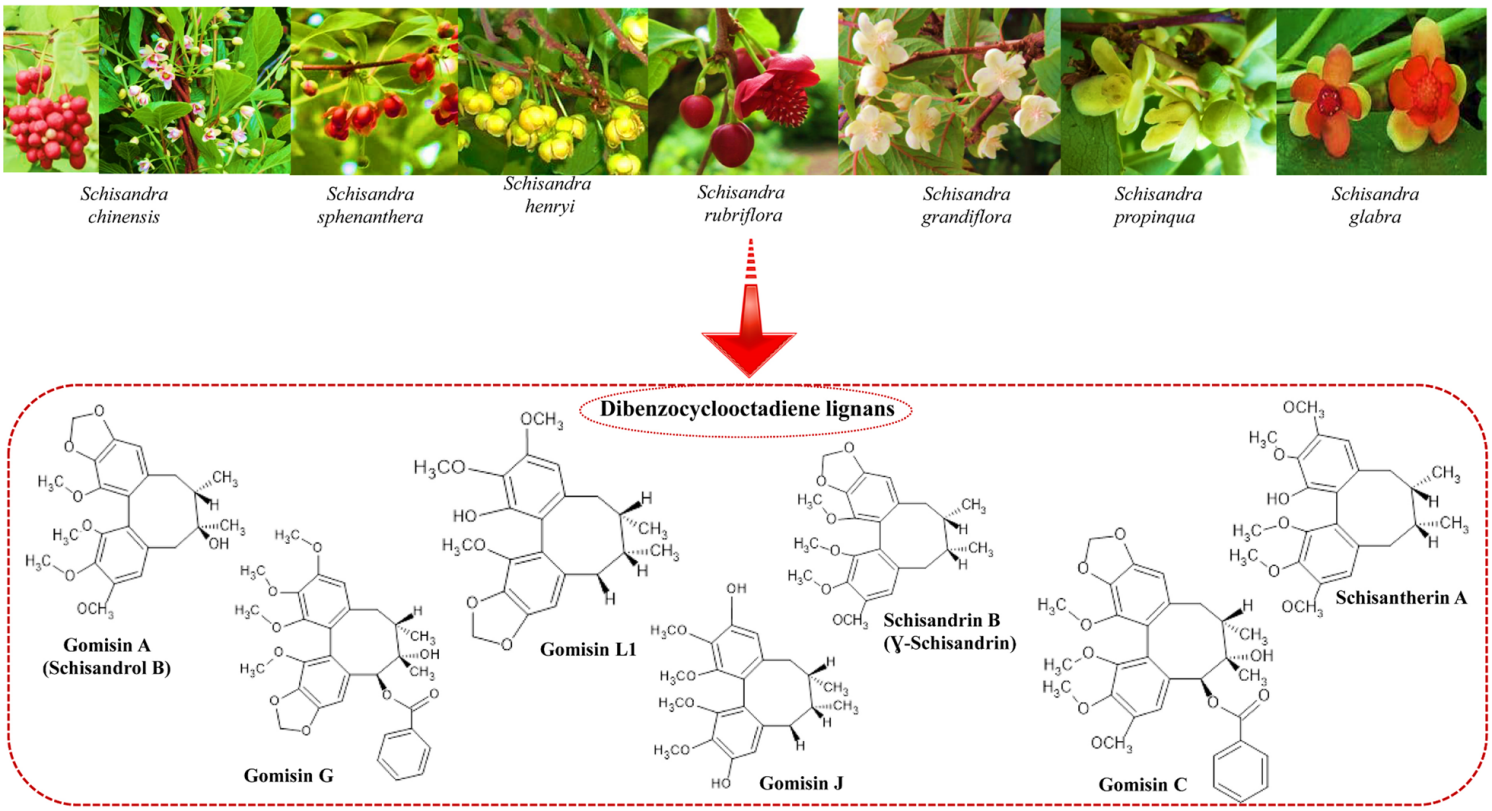
Chemical structures of selected DBCLS of confirmed antitumor potential

DBCLS in chemical classification referred to as type A lignans, are plant secondary metabolites characteristic of the Schisandraceae family, to which about 40 compounds belong [31, 65, 119]. In nature, lignans exist only as one enantiomer or enantiomeric mixtures of different enantiomeric compositions [31]. In scientific studies, the DBCLS synonymous names are often used; ‘*Schisandra* lignans’, or ‘*Schisandrae chinensis* lignans’. The biosynthetic pathway of dibenzocyclooctadiene lignans (Fig. 1) has not been fully understood yet. The likely biosynthetic pathway derives from the conversion: coniferyl alcohol → pinoresinol (furofuran) → lariciresinol (furan) → secoisolariciresinol (dibenzylbutane) → matairesinol (dibenzylbutyrolactone) [119] (Fig. 1). DBCLS do not have 9 (9′)—oxygen attached and can be formed by coupling propenylphenols [120]. The first dibenzyclooctadiene lignin—schisandrin, was isolated from *Schisandra chinensis* seed oil by Kochetkov in 1961 [121]. At present, about 150 lignan derivatives with the dibenzocyclooctadiene skeleton have been isolated [122]. The structures of DBCLS derivatives were confirmed due to their stereostructures with S or R-biphenyl configuration groups [31]. The antitumor activity has been confirmed in the reviewed by us studies, conducted so far 7 DBCLS: gomisin A, gomisin G, schisandrin B, schisanhenol, gomisin L1, gomisin J and schisantherin A [123–127]. The chemical structures of the above-mentioned lignans are presented in Fig. 2.

**Fig. 3:**
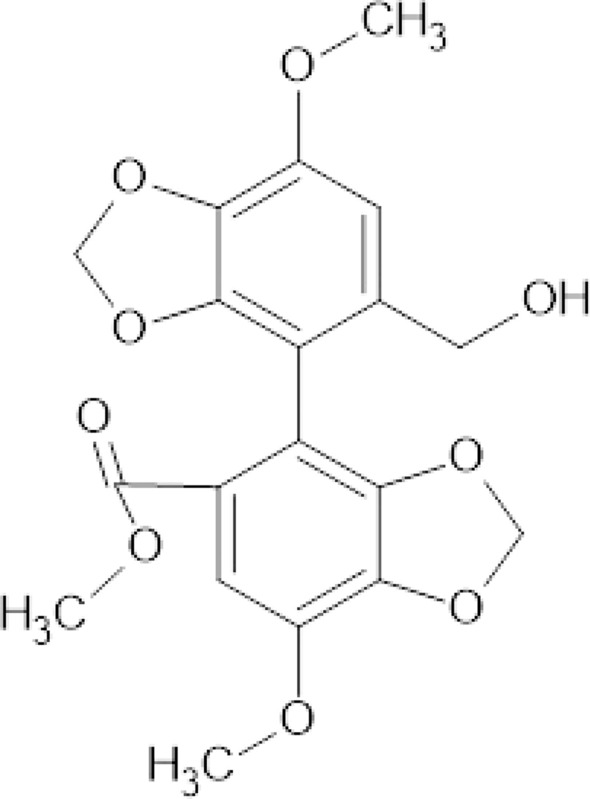
Chemical structure of bicyclol

One of the most popular derivatives of DBCLS is bicyclol (Fig. 3). Chemically, it is an analog of schisandrin C.

## Pharmacokinetics and bioavailability of DBCLS

Understanding the pharmacokinetics of DBCLS is crucial for assessing their therapeutic efficacy and in vivo activity. While key pharmacokinetic parameters such as absorption, distribution, metabolism, and excretion are vital to consider, only limited research has been conducted in this area for DBCLS. In a recent study conducted by Li et al., the researchers aimed to determine the plasma concentration of schisandrin in rats and a validated method was used to achieve this [128]. *S. chinensis* oral single doses were 3 g/kg and 10 g/kg and Equivalent dosages of schisandrin for the two *S. chinensis* product groups were 5.2 mg/kg (3 g/kg) and 17.3 mg/kg (10 g/kg). For absorption rate, Tmax values for oral administration ranged from 22 to 200 min. The pharmacokinetic study included both intravenous treatment (10 mg/kg) and oral administration (10 mg/kg, 3 g/kg, and 10 g/kg) in rats. Parameters like half-life, clearance (CL) 0.09 L/min., mean residence time (MRT), area under the concentration–time curve (AUC) L, and volume of distribution were determined using WinNonlin software and non-compartmental analysis. The key pharmacokinetic parameters for intravenous group were: MRT: 34.80 min, CL: 0.09 L/min, AUC: 43.11 min ng/mL. The AUC values for oral administration: Schisandrin group: 6.71 ± 4.51 min ng/mL; group with 3 g/kg *S. chinensis* product: 17.58 ± 12.31 min ng/mL and for the group with 10 g/kg *S. chinensis* product: 28.03 ± 14.29 min ng/mL. Within the first 120 min, the plasma concentrations of schisandrin from pharmaceutical *S. chinensis* products were notably higher than from the pure compound [128]. The low oral bioavailability of schisandrin may be attributed to hepatic first-pass and intestinal effects. Its rapid metabolism might result in minimal schisandrin entering circulation, hence a low bioavailability at high doses. The Cmax and Tmax of pharmaceutical *S. chinensis* products differ significantly from the single ingestion group. Bioavailability of pure schisandrin versus *S. chinensis* product: pure schisandrin, with a molecular weight of less than 500 Da, dissolves completely in water, leading to a more efficient absorption than its counterpart from extract mixtures; commercial *S. chinensis* products might slow down and delay absorption due to the presence of excipients. Rats given *S. chinensis* displayed schisandrin in their plasma for up to 6 h, with a prolonged retention time compared to pure schisandrin. This extended retention might be attributed to the reabsorption and retention of schisandrin in the blood. The research provides crucial comparative pharmacokinetic information about schisandrin in its pure form versus its presence in pharmaceutical products, contributing valuable insights to clinical applications [128].

## Current preclinical evidence on anticancer molecular mechanisms of DBCLS

### Apoptosis induction

**Fig. 4:**
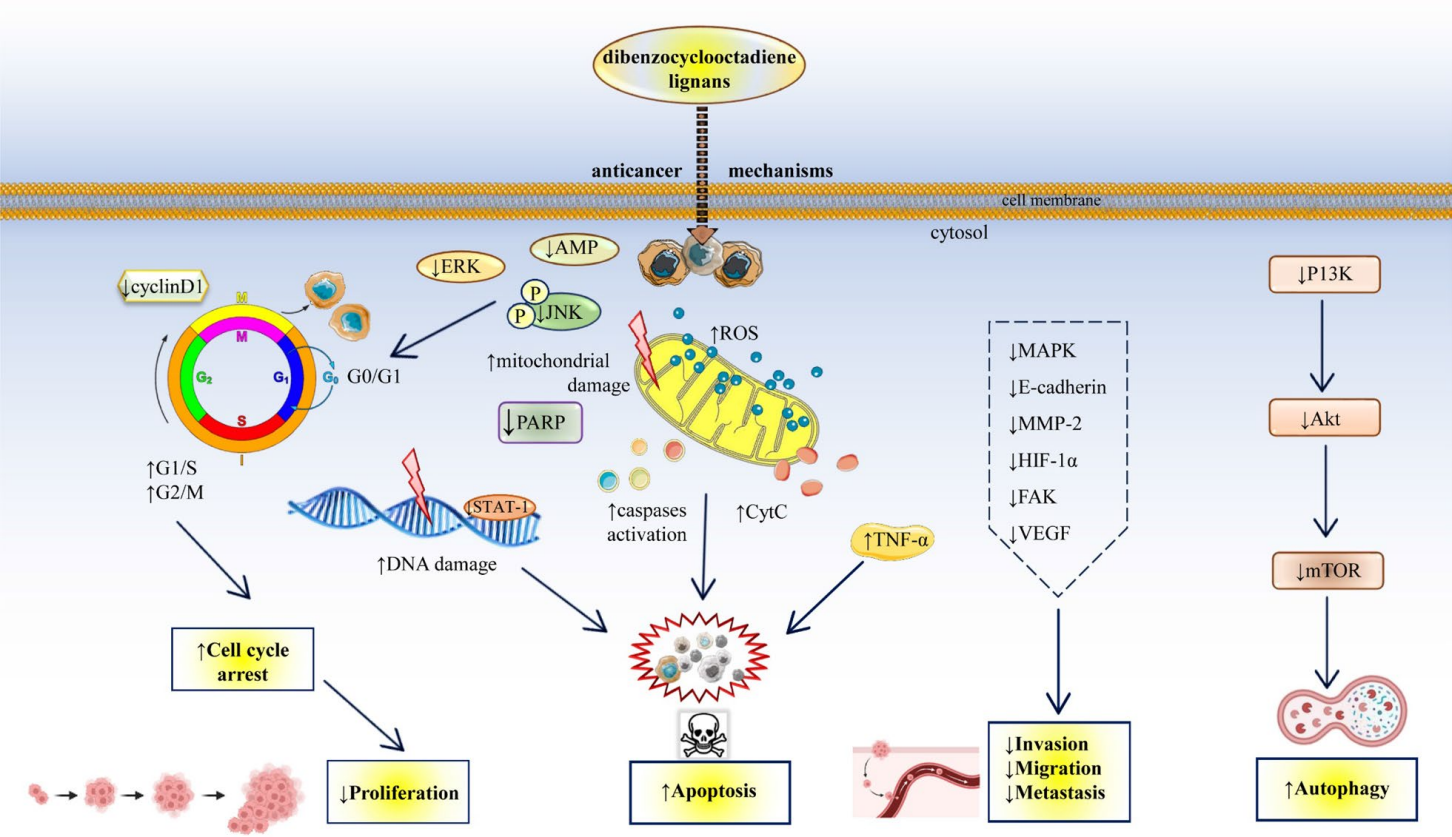
Schematic overview of the principal mechanisms and targeted signalling pathways mediated by DBCLS from the *Schisandra* genus in cancer treatment emphasizing their role in arresting the cell cycle and promoting apoptosis through the mitochondrial pathway and caspase activation. It highlights the induction of cell cycle arrest, particularly in the G1/S and G2/M checkpoints, which leads to a marked decrease in cancer cell proliferation; correspondingly, a significant reduction in cyclin D1 expression is noted, a key regulator of cell cycle progression. Furthermore, DBCLS are shown to enhance apoptotic pathways through mitochondrial perturbations, caspase activation, and cleavage of poly (ADP-ribose) polymerase (PARP), contributing to programmed cell death. They also elevate reactive oxygen species (ROS) within cells, contributing to oxidative stress and potential cellular damage. Simultaneously, the diagram indicates a suppression of the PI3K/Akt/mTOR signaling pathway by DBCLS, which correlates with an increase in autophagic activity, a cellular degradation process that can lead to cell death under certain conditions. Additionally, the ligands inhibit cellular mechanisms responsible for invasion, migration, and metastasis, likely through the modulation of molecules and pathways such as MMP-2, E-cadherin, HIF-1α, FAK, VEGF, and the MAPK pathway. The upregulation of tumor necrosis factor-alpha (TNF-α) further suggests that DBCLS might induce apoptotic signaling pathways. Symbols: ↑ increase, ↓ decrease. *Akt* Protein kinase B, *AMPK* AMP-activated protein kinase, *Cyt C* Cytochrome c, *E-cadherin* Epithelial cadherin, *ERK* Extracellular signal-regulated kinase, *FAK* Focal adhesion kinase, *HIF-1α* Hypoxia-inducible factor 1-alpha, *JNK* c-Jun N-terminal kinase, *MAPK* Mitogen-activated protein kinase, *MMP-2* Matrix metalloproteinase-2, *mTOR* Mammalian target of rapamycin, *PARP* Poly (ADP-ribose) polymerase, *PI3K* Phosphoinositide 3-kinase, *ROS* Reactive oxygen species, *STAT-1* Signal transducer and activator of transcription 1, *TNF-α* Tumor necrosis factor alpha, *VEGF* Vascular endothelial growth factor

The anticancer potential of DBCLS is prominently marked by their capacity to induce apoptosis, a regulated cellular process crucial for eliminating damaged or superfluous cells, thereby maintaining cellular balance; this mechanism is vital in cancer therapy for its role in curbing tumor growth and proliferation [124, 129, 130]. Preclinical studies have demonstrated that DBCLS can trigger apoptosis across various cancer cell lines, predominantly through the mitochondrial apoptotic pathway. This involves the release of cytochrome c (Cyt C) and activation of caspases. While promoting apoptosis is a common mechanism across different DBCLS, these lignans also employ distinct methods to combat cancerous cells. Beyond their role in apoptosis, DBCLS exhibit cytotoxic effects that include inhibiting cancer cell proliferation, invasion, and migration; moreover, individual lignans within this group have been observed to exert anti-inflammatory effects and enhance antioxidant defenses, contributing to their overall anticancer activity [124, 129, 130]. From the particular DBCLS, regarding the number of works on anti-cancer activity, the highest interested is focused on schisandrin B. Its main mechanism of action is inhibition of cell apoptosis induced by TNF-α. Xiang et al. focused on studying the potential use of schisandrin B in treating gallbladder cancer. Tests were performed on the GBC-SD and NOZ gallbladder cancer cell lines. Schisandrin B has been shown to effectively inhibit cancer cells' viability and proliferation and increase apoptosis. Additionally, in vivo tests were performed on nude mice with subcutaneously NOZ tumour xenografts. The results showed that, in this case, schisandrin B also induced apoptosis in cancer cells by regulating the expression of proteins associated with their apoptosis [129] (Table 2). DBCLS can induce oxidative stress in cancer cells by increasing ROS production (Fig. 4). Excessive ROS can damage cellular macromolecules, including lipids, proteins, and DNA, leading to cell death. Ko et al. tested Gomisin L1 as an antitumor compound for ovarian cancer based on the A2780 and SKOV3 cell lines. The results showed that gomisin L1 had a strong cytotoxic effect on cancer cells by inducing apoptosis of cancer cells by regulating the intracellular production of reactive oxygen species and inhibiting NADPH oxidase [130] (Table 2).

**Table 2:** Comprehensive overview of the molecular mechanisms of DBCLS anticancer activity

Anticancer mechanism	Molecular and cellular target	Tested DBCL	Experimental model	Dosage	Anticancer effect	Refs.
Apoptosis induction	Mitochondrial apoptotic pathway (Cytochrome c, Caspases)	Schisandrin B	Human gallbladder cancer GBC-SD and NOZ cell lines	Schisandrin B at 0, 30, 60 and 90 μmol/L	↓ Cell viability ↑ Apoptosis	[129]
Cell cycle arrest	G0/G1, G1/S, G2/M phases	Schisandrin B	Gastric cancer SGC-7901 cell line	Schisandrin B (75 μM), or apatinb (60 μM) with schisandrin B (75 μM)	↓ Cell proliferation ↓Invasion, ↓Migration ↑Efficacy of apatinib	[132]
		Gomisin A	Cervical cancer HeLa cell line Mice C57BL/6 (in vivo) mouse melanoma B16F10 cell line and human melanoma cell line A375SM (in vitro)	Gomisin A at 10, 30 and 100 µM in the presence or absence of TNF-α (20 ng/mL) gomisin A at 2, 10, and 50 mg/kg orally administered to mice once a day, mice were sacrificed after 14 days; gomisin A at 0, 25, 50, 100 µM gomisin A; for 24 h	↓ Cell viability ↓ Migration and invasion	[125, 133]
		Schisantherin A	Human tongue squamous HN4 cell line, macrophage-like RAW264.7 cell line, human gastric MKN45 and SGC-7901 cancer cell lines, human breast cancer MCF7 and MDA-MB-231 cell lines	Schisantherin A at 0 nM, 500 nM, 1 μM and 2.5 μM	↑ Anti-proliferative effect ↑ Cell cycle arrest	[134]
ROS generation	Oxidative stress response	Gomisin L1	Ovarian cancer A2780 and SKOV3 cell lines (in vitro)	Gomsin L1 at 3.12, 6.25, 12.5, 25, 50 and 100 µM	↑ Apoptosis via ROS production	[130]
Autophagy induction	mTOR	Gomisin J	Breast cancer MCF7 and MDA-MB-231, and normal MCF10A cell lines (in vitro)	Gomisin J at 10–30 µg/mL	↑ Autophagy-mediated cell death	[131]
Inhibition of proliferation, invasion, metastasis	MMP activity modulation	Schisandrin B	Prostate cancer DU145 and LNCaP cell lines (in vitro)	Schisandrin B at 0, 50, 100, 150 and 200 µM	↓ Cell growth ↓ Migration ↑ Invasion ↑ Apoptosis	[135]
Chemotherapy resistance modulation	Sensitization to chemotherapy agents, P-glycoprotein expression	Schisandrin B	Breast cancer MDA-MB-435S, MCF-7/ADR, MCF-7 and ovarian cancer A2780 cell lines (in vitro)	Schisandrin B at 10 μM	↑ Sensitivity to doxorubicin ↑ Apoptosis ↓ Tumor growth and migration	[137, 141]
			Cervical cancer (Caski cells) BALB/c nude mice xenografts (in vitro and in vivo)	In vivo experiments Schisandrin B at 20 mg/kg b.w	↓ Cell viability ↓ Colony formation ↑ Apoptosis ↓ Tumor cell invasion ↑ Cytotoxic effect of docetaxel	[142]
		Schisandrin B combined with epirubicin	Breast cancer MDA-MB-435S, MCF-7/ADR, MCF-7 and ovarian cancer A2780 cell lines (in vitro)	Schisandrin B at 10 μM	↑ Cytotoxic effect ↓ Vascular mimicry	[137]
		Schisandrin B	Gastric cancer SGC7901 and BGC823 cell lines (in vitro)	Schisandrin B at 0, 10, 25, 50, 100, 200 μM	↑ Cytotoxic effect by arresting G0/G1 cell cycle	[138]
		Schisandrin B	Gastric cancer SGC-7901 cell line (in vitro)	Schisandrin B at 75 µM	↑ Invasion and migration of apatinib ↑ Cytotoxic drug-induced apoptosis	[132]
		Schisandrin B	Gastric cancer BGC-823 cell line (in vitro) and nude mouse model bearing allograft (in vivo)	Schisandrin B at 0.1 μM (in vitro), 5 mg/kg b.w. (in vivo)	↓ Tumor metastasis; Selective accumulation at tumor site	[140]
Regulation of signaling pathways	MAPK, PI3K/Akt, NF-κB, Wnt/β-catenin	Schisandrin B	Prostate cancer DU145 and LNCaP cell lines (in vitro)	Schisandrin B at 0, 50, 100, 150 and 200 µM	↓ Cell proliferation; ↑ Apoptosis	[135, 143]
		Gomisin G	Breast cancer MDA-MB-231 and MDA-MB-468 cell lines (in vitro)	Gomisin G at 1, 5 and 10 µM		

*ROS* reactive oxygen species, *MMP*: matrix metalloproteinase, *MAPK*: mitogen-activated protein kinase, *PI3K/Akt*: phosphoinositide 3-kinase/protein kinase B, *NF-κB*: nuclear factor kappa B, *mTO*R: mammalian target of rapamycin, *↑* increase, *↓*decrease

### Autophagy induction

Jung et al. examined the anticancer activity of gomisin J. Lignan, which was tested in concentrations 10–30 µg/mL on breast cancer cell lines MCF7 and MDA-MB-231 and in normal MCF10A. Studies have shown that gomisin J has a much stronger cytotoxic effect on cancer cells than on cells of an unaffected line. Gomisin J exhibits a unique ability to modulate autophagy in cancer cells, particularly in MCF7 and MDA-MB-231 lines. Initially, it induces a survival form of autophagy, but with prolonged exposure (over 72 h), this shifts to autophagy-mediated cell death. This effect appears linked to the inhibition of the mTOR pathway, part of the PI3K/Akt/mTOR signaling axis, often utilized by cancer cells for drug resistance. Thus, gomisin J holds potential as a therapeutic agent, especially in treating cancers that have developed resistance to conventional treatments [131]. The ability of DBCLS to induce autophagy in cancer cells represents a significant area of interest, as manipulating this pathway could offer new strategies in cancer therapy.

### Cell cycle arrest

DBCLS compounds can induce cell cycle arrest, particularly at the G1/S and G2/M phases, leading to inhibited cancer cell proliferation (Fig. 4). Li et al. studied the anticancer mechanism of the combination of schisandrin B and apatinib (cytotoxic drug) in in vitro tests on gastric cancer cell lines. A lot of studies have been carried out that have confirmed that such a synergistic combination affects the division of cancer cells by arresting the cell cycle in the G0/G1 phase. Schisandrin B also positively increased the invasion and migration of apatinib in cancer cells and intensified cytotoxic drug-induced apoptosis of cancer cells [132] (Table 2).

Han et al. focused on research on the anticancer effect of gomisin A on melanoma cells. Studies involving the WST test (based on the study of induction and inhibition of cell proliferation in any in vitro model) and flow cytometry were conducted to answer the question of whether gomisin A affects proliferation, apoptosis and cell cycle arrest in melanoma cell lines. In addition, the invasion and migration capacity of melanoma cells was tested in wound healing assays. Gomisin A has been shown to reduce the viability of melanoma cells by inhibiting the cell cycle; the reduction in the expression of the arrest in the G0/G1 phase was diminished following the inhibition of cyclin D1, AMPK, ERK, and JNK phosphorylation. It has also been found to have anti-proliferative effects and reduce cell migration and invasion. Gomisin A inhibited lung metastases by suppressing the epithelial-mesenchymal transition [133]. Waiwut et al. investigated the effect of gomisin A on the division and cell cycle arrest of the HeLA cell line. The results showed that gomisin A significantly inhibited cell proliferation in a dose-dependent manner, primarily in the presence of TNF-α. Gomisin A also significantly affected the arrest of the cell cycle in the G1 phase, causing retinoblastoma cell phosphorylation. In addition, this compound, in synergy with TNF-α inhibited the activity of signal transducer and transcription activation 1 (STAT1) [125] (Table 2). Wang et al. focused on testing the anticancer activity of schisantherin A. The research was conducted on the MKN45 and SGC-7901 gastric cancer cell lines. Schisantherin A has been shown to have a potent anti-proliferative effect on cancer cells by promoting cell cycle arrest in the G2/M phase, inducing tumor cell death and inhibiting cancer cell trafficking. Additionally, schisantherin A promoted JNK phosphorylation, which was dependent on reactive oxygen species, with their overproduction [134], two other trirterpenoid acids—schisandrolic acid and isoschisandrolic acid from *Schisandra propinqua* have anticancer effects. The cytotoxic research of these compounds shows that both have a strong cytotoxic effect on HepG2 cell lines by inhibiting the G0/G1 phases and then inducing the death of tumour cells; additionally, this process is accompanied by the proteolytic cleavage of poly-ADP-ribose polymerase (PARP), a substrate for caspases, which is associated with the onset of apoptosis in HepG2 cells [118].

### Inhibition of cancer cell proliferation, invasion and metastasis

Several studies have suggested that DBCLS compounds can inhibit cancer cell migration and invasion (Fig. 4). This might be attributed to their ability to modulate matrix metalloproteinase (MMP) activity, which plays a crucial role in tissue remodelling during cancer metastasis. Another DBCL compound with confirmed anticancer activity is gomisin A [133]. This compound primarily uses the mechanism of action consisting of stopping the cell cycle and death of cancer cells but also reduces their migration and invasion. Gomisin A also has a beneficial effect on inhibiting the proliferation of cancer cells by inhibiting the expression of the signal transmitter and tumour necrosis TNF-α. Schisandrin B also inhibits cancer cell growth, migration and invasion by increasing the number of heat shock proteins. A large amount of research is also focused on combining schisandrin B with other substances that inhibit the migration of cancer cells [135, 136]. Jing et al. focused on more advanced research involving combining schisandrin B with a PFV-modified epirubicin cytotoxic drug. In addition, schisandrin B was encapsulated in liposomes. The studies were based on the MDA-MB-435S breast cancer cell line. Tests on this cell line have shown that the resulting complex enhances cytotoxicity, affects the development of vascular mimicry and inhibits tumour invasion and spread. The mechanism of action against cancer cells is based on the regulation of the expression of vimentin, E-cadherin, vascular endothelial growth factor (VEGF) and matrix metalloproteinase-9 (MMP-9). Tests were also carried out on mice. Studies on living organisms have shown that the complex can increase the apoptosis of cancer cells [137] (Table 2). He et al. also focused on the effects of schisandrin B on gastric cancer cells. The tests were carried out both in vivo and in vitro. In addition to schisandrin B independent activity, combining this lignan with 5-fluorouracil (a cytotoxic drug) has also been studied. Studies have shown that schisandrin B completely inhibited cancer cell development, proliferation and invasion. Additionally, schisandrin B was found to increase the efficacy of 5-fluorouracil, induce apoptosis, and inhibit STAT3 phosphorylation [138] (Table 2). Wang et al. tested the anticancer effect of schisandrin B on 143B, MG63, Saos2 and U2OS osteosarcoma cell lines in vitro and in vivo and the mechanism of action of the lignan. In tests, schisandrin B effectively inhibited cancer cells’ migration, invasion and multiplication. Additionally, schisandrin B was found to promote apoptosis of osteosarcoma cells. Studies have also been carried out on healthy cells, which have shown that schisandrin B has no negative effect on the viability of these cells. By examining the mechanism of action of schisandrin B, it was found to inhibit the cell cycle's proliferation in the G1 phase [139]. Casarin et al. focused on testing two DBCLS: deoxyschisandrin and schisandrin B. Lignans were tested on two cancer cell lines 2008 and LoVo; both compounds showed an inhibitory effect on the proliferation and development of cancer cells but differed in the mechanism of action—deoxyschisandrin induced apoptosis in the LoVo cell lines but not in the 2008 line; schisandrin B caused apoptosis in both cell lines. It was found that the pathway in which cell organelles—mitochondria participate—did not participate in the apoptosis of cancer cells (25) (Table 1). For gomisin L1, gomisin J and schisantherin A, it is assumpted that the main mechanism of their action is the induction of cancer cell death [131, 134]; additionally, gomisin J and schisantherin A inhibited their proliferation invasion and viability [131]. Li et al. also used liposomes in their studies, in which they encapsulated vinoreblin (cytotoxic drug), R8 peptide and schisandrin B in order to study the activity against gastric cancer cells. Peptide R8 was used for its cellular uptake-enhancing effect, schisandrin B for metastasis inhibition, and vinoreblin was used as a chemotherapeutic agent. Experiments were performed on the human gastric cancer cell line BGC-823. It was shown that the liposomal complex induced apoptosis of cancer cells by reducing the level of VE-Cadherin, PI3K, VEGF, HIF-1α, FAK and MMP-2, which resulted in the inhibition of invasion and metastasis. In addition, in vivo tests showed that liposomes selectively accumulated in places occupied by cancer cells and induced their cellular death [140] (Table 2).

### Chemotherapy resistance modulation

Multi-drug resistance is a significant obstacle in cancer treatment. Preliminary studies have suggested that DBCLS could enhance the sensitivity of cancer cells to conventional chemotherapy agents, making them more effective (Fig. 4). A combination of schisandrin B with the powerful cytotoxic drug doxorubicin was tested. Studies have shown that schisandrin B increases the sensitivity of cancer cells to the drug and induces their apoptosis. Doxorubicin derivatives—epirubicin—were also used in the study [137]. The combination of both substances increased the cytotoxic effect, destroyed the formation of vascular mimicry and inhibited the growth and migration of the tumour. Also, the action of schisandrin B in combination with 5-fluorouracil and schisandrin B with apatinib showed a cytotoxic effect on cancer cells by arresting the G0/G1 cell cycle [132]. Schisandrin B, combined with vinorelbine encapsulated in liposomes, also showed promising results regarding anticancer activity—they inhibited tumour metastasis by selectively accumulating at the target site [140]. Wang et al. tested schisandrin B as a treatment for doxorubicin-resistant cancer. Breast and ovarian cancer cell lines were tested. Studies have shown that schisandrin B significantly increased the intracellular accumulation of doxorubicin through an inhibitory mechanism of action on P-glycoprotein expression and activity. Additionally, schisandrin B downregulated the anti-apoptotic survivin expression [141] (Table 2). Yan et al. focused on studying the anticancer effect of schisandrin B on cervical cancer synergistically combined with docetaxel, which, as a chemotherapeutic agent, limits the growth and invasion of the cervical tumour. The studies were performed on Caski cells—a positive cell line that serves as a model for analysing advanced cervical carcinoma. The results show that schisandrin B and docetaxel reduced cell viability, inhibited colony formation, induced apoptosis and inhibited tumour cell invasion. Administration of both compounds at the same time intensified these processes. Additionally, in vivo studies were performed in BALB/c nude mice xenografted with Caski cells. The results of these experiments showed that the synergistic combination of schisandrin B and docetaxel, similar to in vitro studies, enhanced the effect of docetaxel by influencing the mechanisms described above [142] (Table 2).

### Regulation of signaling pathways

DBCLS compounds can modulate various signalling pathways involved in cell survival, proliferation, and death. Examples include the MAPK, PI3K/Akt pathway, and NF-κB pathway. Nasser et al., based on earlier studies on schisandrin B, which confirmed the cytotoxic effect of this lignan on prostate cancer cells, decided to focus on the mechanism of schisandrin B anticancer activity. The research was carried out on the DU145 and LNCaP prostate cancer cell lines. Schisandrin B has been found to inhibit the proliferation and induce apoptosis of cancer cells. In addition, it inhibits their cell cycle in the S phase. Detailed tests on the induction of apoptosis by schisandrin B suggest that the whole process is related to the lignan's ability to generate oxidative stress in cancer cells and the inhibitory effect on the androgen receptor and PI3K/AKT and STA3/JAK2 phosphorylation [135]. In vivo tests in animal models have shown that schisandrin B has an inhibitory effect on cell division, migration and invasion. Additionally, schisandrin B induces apoptosis by stopping the Wnt/β-catenin and PI3K/Akt signalling pathways while showing no deleterious effects on normal cells [139] (Table 2). Maharjan et al. tested the anticancer effect of gomisin G on the so-called triple-negative breast cancer, which has a lower survival rate. The studies were conducted on two cell lines MDA-MB-231, MDA-MB-468, MCF-7, T47D and ZR75-1. It was shown that gomisin G effectively inhibited the viability of cancer cells from the MDA-MB-231 and MDA-MB-468 lines. The mechanism of action of gomisin G was based on a very high inhibitory activity on AKT phosphorylation and a decrease in the amount of retinoblastoma tumour suppressor protein and phosphorylated retinoblastoma tumour. The action of gomisin G also focused on inhibiting the amount of cyclins D1 and inhibiting the cell cycle in the G1 phase [143] (Table 2).

Table 2 provides a detailed summary of DBCLS anticancer activity, encompassing various mechanisms, molecular targets, compounds tested, experimental models, dosages, anticancer effects.

## DBCLS as potential chemotherapeutic adjuvants

### Bicyclol: therapeutic effects and anticancer potential

In 2004, the China Food and Drug Administration (CFDA) licensed bicyclol, a derivative of DBCLS for drug marketing and its production [144, 145]. This compound has found strong potential in diseases related to the liver, in particular inflammation, including viral hepatitis type B and C. A large number of studies have focused on confirming the hepatoprotective effect on liver cells with positive results. The mechanism of action used by bicyclol is primarily the inhibition of hepatocyte apoptosis associated with a number of signalling pathways that promote the expression of hepatic heat shock proteins. A large number of clinical studies have been conducted on the effects of bicyclol on the body and bicyclol has been shown to cause no noticeable side effects [144, 145]. In addition to many studies that focus primarily on the hepatoprotective effect of bicyclol, researchers are looking for other biological activities of this compound, including anticancer activity. Researchers focused on the mechanism of inhibition of the development of HepG2 hepatoma cells. It has been shown that bicyclol has an inhibitory effect on the proliferation of HepG_2_ cells by using the mechanism of inhibition of the cell cycle in the G1 phase and the initiation of autophagy in cancer cells. In addition, it was shown that bicyclol stopped the phosphorylation of ERK and Akt and effectively reduced the expression of cyclin E2, cyclin D1, CDK4, CDK2, p-Rb and p-mTOR [146]. Research also focused on understanding the effect of bicyclol on renal cell carcinoma. The results show that bicyclol favourably affects the initiation of apoptosis of cancer cells and stops their cell cycle. The compound also increased the synthesis of reactive oxygen species, which increased oxidative stress in cancer cells [147]. There have also been very advanced studies on bicyclol in vivo as an effective anticancer agent in treating hepatocellular carcinoma and hepatoma. The tests were performed on mice with liver cancer induced by diethylnitrosamine and promoted by phenobarbital. Liver tissue and mouse model serum were collected for testing. The research was divided into two stages. In the first step, mice were given bicyclol orally before diethylnitrosamine injection. The results showed a significant reduction in the inflammatory infiltration of tumours in the liver. Mice treated with bicyclol at week 20 were found to be free of tumour cells after histopathological examination. In addition, after 10 weeks without diethylnitrosamine and phenobarbital, bicyclol did not develop a hepatoma. Compound-induced mice not given bicyclol developed hepatoma in 100% and hepatocellular carcinoma in 50% [148]. Another study also focused on the anticancer effect of bicyclol on liver cancer. The tests were performed on ICR mice, which were orally administered bicyclol 2 h before intraperitoneal injection of cisplatin (hepatotoxic substance) for 5 days after H22 cell implantation. The results indicate that bicyclol has a hepatoprotective potential. It has been shown that it caused a decrease in the activity of serum transaminases and lactate dehydrogenase and positively affected the reduction of cisplatin-induced liver tissue damage. In addition, bicyclol inhibited the production of hepatic malondialdehyde [149]. Subsequent studies have confirmed that bicyclol has a supportive effect in the treatment of cancer, primarily by reducing the resistance of cancer cells to chemotherapeutic agents. It was also confirmed that bicyclol has a supportive effect in the therapy with cytostatic preparations in multidrug resistance (MDR) cancer cell lines resistant to vincristine. Bicyclol has also shown supportive effects in the treatments of VinRKB human cancer, in which the cancer cells did not respond to vincristine, and in the treatment of AdrRMCF-7 breast cancer that is resistant to adriamycin. Detailed studies found that bicicol increases the concentration inside the tumour cell, which affects the sensitisation of cells to the effects of drugs [150]. Other studies have also confirmed the effect of bicyclol on cancer cells. At doses that did not show toxicity, it inhibited the malignant transformation of WB-F344 cells initiated by 3-methylcholtrenne and phorbol 12-o-tetradecanoyl-13-acetate. The mechanism of action of bicyclol consisted of inhibiting the division of cancer cells, which were stimulated by phorbol 12-o-tetradecanoyl-13-acetate, and inhibited the expression of cPKC-α and P-ERK1/2 promoted by the same compound [151].

### Wuzhi capsules and their role in enhancing chemotherapy

In Asian countries, especially in China, the medicinal preparation called Wuzhi capsules is used on a large scale. It is a standardized *Schisandra sphenatnhera* extract containing: schisandrin, schisandrol B, schisantherin A, schisanhenol and deoxyschisandrin. The preparation is used in ailments related to abnormal liver function, such as chronic hepatitis or dysfunction of this organ. Numerous scientific studies confirm the hepatoprotective effect of DBCLS, but at present, researchers are focusing on the new directions of possible use of DBCLS. The studies most often involve the combination of approved immunosuppressive and anticancer drugs with strong side effects with Wuzhi. Fu et al. researched combining the effects of Methotrexate with the Wuzhi capsules. The effect of Wuzhi capsules on the pharmacokinetics of an anticancer drug was tested. It was found that when both substances were administered simultaneously, Wuzhi capsule decreased CLz/F and Vz/F of methotrexate and increased Cmax. In addition, the administration of *S. sphenanthera* extract inhibited the expression of OAT1/3 proteins in the kidneys and P-gp in the small intestine, which may suggest the future use of this combination to reduce inflammation when administering immunosuppressive drugs [152]. The latest research shows that in the future, the combination of the Wuzhi capsule with anti-cancer drugs will be widely available and recommended. Chen et al. focused on combining the Wuzhi capsule with cyclophosphamide—an anti-cancer drug with several side effects, primarily nephrotoxic and hepatotoxic. In tests, the Wuzhi capsule is supposed to attenuate the side effects of the drug. The tests were based on examining the effect of two components of Wuzhi capsules—schisandrin A and schisantherin A on the pharmacokinetics of the chemotherapeutic agent. With co-administration, the AUC of cyclophosphamide was found to increase by 18% and 1% with single doses of schisandrin A and schisantherin A and increase by 301% and 29% with multiple doses of lignans. Cmax of cyclophosphamide was increased by 75% and 7% with multiple lignan administration. The results suggest that Wuzhi capsules can effectively reduce the effect of anti-cancer drugs when administered synergistically [153] Wuzhi capsules manufactured by Fanglue Pharmaceutical Company (Kuangxi, China) contain a standardized dried fruit extract containing 7.5 mg of schisantherin A. In a recent study, Chinese researchers decided to test the effect of the Wuzhi tablet on three important drugs: paclitaxel cyclosporine A and tacrolimus. Tests on animals (rats) have shown that co-administration of Wuzhi tablets with cyclosporine A significantly affects the cyclosporine level in the blood. Presumably, cyclosporine may be sensitive to using CYP3A/P461 gp inhibitors or inducers [154]. Subsequent tests focused on the co-administration of Wuzhi tablets with paclitaxel showed increased blood paclitaxel levels after oral administration, resulting in increased systemic exposure to the drug [66]. Another study of administering Wuzhi tablets and tacrolimus to rats showed that the concentration of tacrolimus in the blood slightly increased [155]. The study also focused on the effect of *S. sphenanthera* fruit extract on absorption and found first-pass metabolism in the gut and liver. It was found that the *S. sphenanthera* fruit extract may increase the oral bioavailability of tacrolimus [155]. A collated summary of research findings on the therapeutic applications and drug interactions of DBCLS from the *Schisandra* genus is presented in Table 3.

**Table 3:** Overview of therapeutic uses and drug interactions of market-approved DBCLS from the *Schisandra* genus

Study Focus	Tested compound/ Drug	Methodology	Major Findings	References
Hepatoprotective effects	Bicyclol	Inhibition of hepatocyte apoptosis	No noticeable side effects Increases hepatic heat shock proteins	[149, 150]
Anticancer activity on HepG2 cells		Cell cycle inhibition autophagy initiation	Inhibits HepG2 cell proliferation, Decreases levels of several key proteins involved in cell cycle and proliferation	[151]
Anticancer activity on renal cancer/carcinoma cell		Apoptosis initiation Cell cycle arrest	Increases oxidative stress in cancer cells	[152]
Hepatocellular carcinoma		Mice model	Significant reduction in liver tumors; 100% hepatoma and 50% hepatocellular carcinoma in control group	[153]
Hepatoprotective and anticancer		ICR mice model with cisplatin	Reduces cisplatin-induced liver tissue damage. Enhances liver enzymes	[154]
Multidrug resistance		In vitro studies	Reduces resistance to vincristine and adriamycin in cancer cell lines	[155]
Anti-transformative effects		Inhibition of WB-F344 cell transformation	Inhibits malignant transformation of cells	[151]
Combination therapies	Wuzhi capsules	Co-administered with methotrexate	Affects Methotrexate pharmacokinetics, suggesting potential to reduce inflammation in immunosuppressive therapies	[152]
Nephrotoxic and hepatotoxic effects		Co-administered with cyclophosphamide	Potentially attenuates side effects of cyclophosphamide Alters pharmacokinetics to increase drug efficacy	[153]
Drug interactions		Co-administered with cyclosporine A, paclitaxel, tacrolimus	Affects blood levels of administered drugs May influence absorption and first-pass metabolism, increasing the oral bioavailability of tacrolimus	[154, 155]

## Limitations, challenges and clinical pitfalls of DBCLS in oncology

While DBCLS from the *Schisandra* genus has shown promising anticancer properties in vitro and in vivo models, no studies have progressed to clinical trials in humans. This limitation restricts our understanding of the lignans' safety, dosage, and therapeutic efficacy in humans. Additionally, a comprehensive understanding of these lignans' metabolism, distribution, excretion, and mechanism of action in the human body still needs to be developed. The long-term side effects, interactions with other medications, and overall safety profile of these lignans in cancer patients haven’t been thoroughly studied. Anticancer agents need to target cancer cells without affecting normal cells. Therefore, the specificity and selectivity of these lignans towards different types of cancer cells versus normal cells remain a challenge and need more comprehensive study. Also, as with many natural bioactive compounds, the bioavailability of these DBCLS might be limited when ingested or administered. This can influence therapeutic outcomes, as the compound needs to reach the tumor site in effective concentrations. Cancers are not a single disease but a group of related diseases. It’s challenging to develop an effective single agent against the myriad of cancer types and their subtypes. Therefore, the lignans' effectiveness across this spectrum remains to be thoroughly evaluated. Some cancer cells develop resistance over time to many anticancer compounds, and it's unknown if prolonged treatment with these lignans will lead to the emergence of resistant cancer cell populations. Efficiently delivering these lignans to the tumor site, especially for solid tumors, remains a technical challenge. Advanced drug delivery systems might be required to improve the therapeutic potential of these compounds. These lignans’ potential synergistic or antagonistic effects when combined with standard chemotherapy or other anticancer agents have yet to be fully explored. Given that these lignans are derived from natural sources, there are inherent challenges in ensuring consistent potency and quality. Other bioactive compounds in the extract may also influence the anticancer activity. A significant clinical gap is in the understanding of DBCLS mechanisms beyond apoptosis, particularly concerning alternative forms of cell death such as necrosis, anoikis, ferroptosis, and pyroptosis. The effects of DBCLS on tumor angiogenesis and the tumor immune microenvironment are also not well-documented, presenting another critical area for future research. This lack of data limits the complete comprehension of the anticancer mechanisms of DBCLS.

## Conclusion and future perspectives

DBCLS from the* Schisandra* genus in oncology have opened new avenues for cancer treatment and prevention. Recognized for their diverse biological activities, DBCLS have been primarily associated with apoptosis induction, cell cycle interruption, and enhancing the effectiveness of existing cytotoxic drugs. This promising potential positions DBCLS as valuable candidates for future oncological applications. The journey from laboratory to clinic remains a pivotal path for DBCLS. Future endeavors should concentrate on human clinical trials to validate these compounds' effectiveness and safety, thereby bridging a significant gap between preclinical findings and clinical applications. While apoptosis induction is a known action of DBCLS, expanding research to include their effects on other cell death modalities, such as necrosis, anoikis, ferroptosis, and pyroptosis, is crucial; this expansion could provide a holistic understanding of DBCLS’s anticancer mechanisms. Also, detailed molecular studies are needed to unravel the complex interactions and pathways influenced by DBCLS. This knowledge could lead to the development of more precise and targeted cancer therapies. In the context of increasing drug resistance in cancer therapy, DBCLS could play a transformative role. Investigating their potential in synergistic therapies with standard chemotherapy agents may offer new strategies to combat resistant cancer forms. In conclusion, DBCLS from the *Schisandra* genus hold substantial promise for advancing oncology treatments. Their multifaceted nature, combined with emerging production methods, paves the way for innovative, natural, and effective cancer therapies. Continued research in these areas is imperative to fully harness and optimize the anticancer potential of DBCLS.

### Supplementary Information

**Additional file 1.** Potential biotechnological studies on Schisandra species in vitro cultures.

## Data Availability

Not applicable.

## Abbreviations

143B: Human osteosarcoma cell line
15-LOX: 15-Lipoxygenase
ADME: Absorption, distribution, metabolism, and excretion
AUC: Area under the curve
BALB/c: Albino, laboratory-bred strain of the house mouse
BGC-823: Human gastric cancer cell line
C8166: Clone of C63/CRII-4 derived by fusion of primary umbilical cord blood cells with HTLV-1 producing line from adult T cell leukaemia lymphoma patient
CaSki: Human papillomavirus type 16 (HPV-16)-positive cell line
CDK2: Cyclin-dependent kinase 2
CDK4: Cyclin-dependent kinase 4
CFDA: China Food and Drug Administration
CosIng: COSMETIC ingredient database
COX-1: Cyclooxygenase-1
COX-2: Cyclooxygenase-2
cPKC-α: Protein kinase C-α
CUPRAC: CUPric Reducing Antioxidant Capacity
CYP3A/P461 gp: Cytochrome 3A/p461 and glycoprotein
Cyt C: Cytochrome C
Da: Dalton
DBCLS: Dibenzocyclooctadiene lignans
DNA: Deoxyribonucleic acid
DPPH: 2,2-Diphenyl-1-picrylhydrazyl
DU145: Human prostate cancer cell line
EFSA: European Food Safety Authority
EMA: European Medicines Agency
FAK: Focal adhesion kinase
FDA: U.S. Food and Drug Administration
FRAP: Fluorescence recovery after photobleaching
GBC-SD: Human gallbladder cancer cell line regulated by TGF-β-induced epithelial-mesenchymal transition
H22: Hepatocellular carcinoma cell line
H2O2: Hydrogen peroxide
HeLa: Cervical cancer cell line
HepG2: Human hepatocellular carcinoma cell
HIF-1α: Heterodimeric transcription factor hypoxia-inducible factor 1 α
HIV-1: Human immunodeficiency virus-1
HPV-16: Human papillomavirus type 16
IC50: Half maximal inhibitory concentration
JNK: C-Jun N-terminal kinase
K562: Human erythromyeloblastoid cell line
LNCaP: Androgen-sensitive human prostate adenocarcinoma cell line
LoVo: Colon adenocarcinoma cell line
MAPK: Mitogen-activated protein kinase
MCF-7: Human breast cancer cell line with estrogen, progesterone and glucocorticoid receptors
MDA-MB-231; -468: Triple-negative breast cancer cell lines
MDA-MB-435S: Breast cancer cell line
MG-63: Human osteosarcoma cell line
MIC: Minimum inhibitory concentration
MMP: Matrix metalloproteinase
MMP-2: Matrix metalloproteinase-2
MMP-9: Matrix metalloproteinase-9
MRT: Mean residence time
NADPH: Nicotinamide adenine dinucleotide
NF-κB: Nuclear factor kappa B
NOZ: Human gall bladder carcinoma cell line
OAT1/3: Organic anion transporter 3
P-388: Monocyte/macrophage-like cell line from a mouse with lymphoma
PC12: Rat adrenal pheochromocytoma cell line
P-ERK1/2: Extracellular signal-regulated protein kinases 1 and 2
PFV: Prototypic foamy virus
P-gp: P-glycoprotein
PI3K/akt: Intracellular signaling pathway in regulating the cell cycle
p-mTOR: Phosphorylated mammalian target of rapamycin
p-Rb: Retinoblastoma protein
R8 peptide: Cell-penetrating peptide
ROS: Reactive oxygen species
Saos2: Human osteosarcoma cell line
SGC-7901: Human gastric cancer cell line
SIHA: Squamous cell carcinoma
sPLA2: Phospholipase A2
STA3/JAK2: Janus kinase 2/signal transducer and activator of transcription 3 pathway
STAT1: Signal transducer and activator of transcription 1
STAT3: Signal transducer and activator of transcription 3
T47D: Human breast cancer cell line
TCM: Traditional Chinese medicine
Tmax: Maximal time
TNF-α: Tumor necrosis factor α
U2OS: Human osteosarcoma cell line
VE-Cadherin: Vascular endothelial cadherin
VEGF: Vascular endothelial growth factor
VSMC: Vascular smooth muscle cell
Vz/F: Apparent volume of distribution during terminal phase
WB-F344: Rat liver progenitor cell line
WHO: World Health Organization
Wnt/β-catenin: Wnt/β-catenin signaling pathway
ZR75-1: Human breast cancer cell line
