# Supplementary material for: Comprehensive review of dibenzocyclooctadiene lignans from the Schisandra genus: anticancer potential, mechanistic insights and future prospects in oncology
Source: Chin Med. 2024 Jan 24;19:17. doi: 10.1186/s13020-024-00879-0 (PMC10809469; doi:10.1186/s13020-024-00879-0)
Supplement: Supplementary file 1 — Additional file 1. Potential biotechnological studies on Schisandra species in vitro cultures. [file 13020_2024_879_MOESM1_ESM.docx]

**Additional file**

**Comprehensive review of dibenzocyclooctadiene lignans from the *Schisandra* genus: anticancer potential, mechanistic insights, and future prospects in oncology**

Karolina Jafernik^1^, Sara Motyka^1,2^, Daniela Calina^3,*^, Javad Sharifi-Rad^4,*^, Agnieszka Szopa^1,*^

^1^Chair and Department of Pharmaceutical Botany, Jagiellonian University, Medical College, Medyczna 9 St., 30-688 Kraków, Poland; karolina.jafernik@doctoral.uj.edu.pl (K.J.), a.szopa@uj.edu.pl (A.S.)

^2^Doctoral School of Medical and Health Sciences, Medical College, Jagiellonian University, Łazarza 16 St., 31-530 Kraków, Poland; sara.motyka@doctoral.uj.edu.pl (S.M.)

^3^Department of Clinical Pharmacy, University of Medicine and Pharmacy of Craiova, 200349 Craiova, Romania; calinadaniela@gmail.com (D.C.)

^4^Facultad de Medicina, Universidad del Azuay, Cuenca, Ecuador

*Corresponding authors: Daniela Calina ([calinadaniela@gmail.com](mailto:calinadaniela@gmail.com)); Javad Sharifi-Rad ([javad.sharifirad@gmail.com](mailto:javad.sharifirad@gmail.com)); Agnieszka Szopa (a.szopa@uj.edu.pl)

**Potential biotechnological studies on *Schisandra* species in vitro cultures**

***Schisandra chinensis***

Research on *in vitro* cultures of *S. chinensis* in the field of microreproduction and endogenous production of secondary metabolites was carried out in research centers in the countries of East Asia: China, Japan and South Korea. In European countries, researches have been carried out in the Czech Republic and Poland. Productions of lignans in the biomass of *S. chinensis* *in vitro* cultures was investigated by Havel et al. [1] and Březinová et al. [2]. The analysis confirmed the presence of compounds from the DBCLS group: Ɣ-schisandrin and schisantherin A. The presence of gomisin A, gomisin N and Ɣ-schisandrin was also confirmed in embryonic agar cultures kept in the dark and in suspension cultures carried out in the same photoperiod. In the analyzed extracts, the dominant compound was gomisin N - max. 0.55 g/100 g DM (dry mass), while in extracts from suspension cultures it was Ɣ-schisandrin - max. 0.54 mg/100 g DM. For comparative purposes, lignan concentrations in leaves of *S. chinensis* growing *in vivo* were analysed and the gomisin A was the main compound - max. 0.8 mg / 100g DM [1,2]. Kohda et al. studied callus cultures of *S. chinensis* in which the dominant was gomisin A (0.05% of callus dry weight) [3]. Szopa et al. conducted studies on the production of DBCLS in *in vitro* microshoot cultures of *S. chinensis.* The highest amounts were obtained for gomisin A - max. 86 mg/100 g DM; and it was higher than in extracts from fruits and leaves of parent plant [4,5]. Further studies on the accumulation of DBCLS in biomass from *S. chinensis in vitro* cultures showed that the extracts contain a high content of schisantherin A - max. 33 mg/100 g DM and gomisin G - max. 22 mg/100 g DM) [5,6]. Szopa et al. also conducted studies on the influence of the microshoot cultivation mode on the accumulation of DBCLS. Agar, liquid stationary and agitated cultures were tested. Dominant compound in all types of cultures was gomisin A - max. 34 mg/100 g DM, and the obtained total amounts of DBCLS from extracts from *in vitro* cultures were comparable to leaf extracts and 3.2 times lower than in fruit extracts [7]. Additionally, Szopa et al. focused on studying the effect of monochromatic light on the accumulation of DBCLS in *S. chinensis* microshoot cultures. Cultures on agar medium were carried out in the presence of monochromatic fluorescent light: blue, UV-A, red and far-red. Cultures maintained under white and dark lighting conditions served as control. It was shown that the total amount of lignans increased 1.7 times depending on the light conditions. Blue light turned out to be the most favoring the DBCLS production - the total concentration of lignans was 376 mg/100 g DM, and the dominant compound was gomisin A (max. 37 mg/100 g DM) [8]. Szopa et. al. also proved that elicitation with methyl jasmonate (MeJa), yeast extract (YeE), cadmium chloride (CdCl_2_), chitosan (Ch), and the adition of the permeabilizing agent - dimethyl sulfoxide (DMSO) influenced on the DBCLS production in *S. chinensis* microshoot cultures. The results showed that the compounds used had a significant effect on the accumulation of lignans in the tested biomass extracts. The best results were obtained after elicitation with YeE 1000 mg/l on the 20th day of culture (max. content of DBCLS was equal 640 mg/100 g DM). The elicitation method presented above was also applied in temporary immersion systems (TIS) - Plantform bioreactors, where the total content of lignans was 830 mg/100 g DM [9]. Szopa et al. elaborated also the possibilities of large scale production of DBCLS in various types of plant bioreactors. The five bioreactor were tested: column bioreactors with a microshoot immobilization system, balloon bioreactors, spray bioreactors and two TIS bioreactors: RITA^®^ and Plantform. The highest production of lignans was found in biomass extracts of cultures grown in Plantform TIS over 30 days - 547 mg/100g DM, and the dominant compound was gomisin A - max 68 mg/100 g DM [9–11].

***Schisandra sphenanthera***

*In vitro* cultures of *S. sphenanthera* are of interest to scientific units only in China [12,13]. This is probably related to the problem of obtaining plant material for researchers who work on this species outside of Asia. As of today, only three reports were published and they are very basic with no information of DBCLS production in the in vitro tissue. Liang et al. were shown that the LS, ½ MS and MS media were optimal for the growth of callus cultures, in which the medium ½ MS was the most conducive to the increase in the production of polysaccharides [13]. Wu et al. showed that MS medium supplemented with 0.1 mg/L NAA and 1.0 mg/L 6-BA and 0.5 mg/L 2,4-D (2,4-dichlorophenoxyacetic acid) turned out to be the best medium for callus induction from *S. sphenanthera* leaves as explants [14]. Wang et al. indicated that MS medium with 0.1 mg/L of NAA and 0.5 mg/L of 2,4-D was the best for the high microshoot induction survival rate of *S. sphenanthera* [12].

***Schisandra henryi***

Jafernik et al. conducted the first biotechnological studies on the *S. henryi* species and provided very promising results with regard DBCLS production using biotechnological methods. The maximal total DBCLS content was max. 873.71 mg / 100 DM. The dominant compound were schisantherin A (max. 143.74 mg/100 g DM) and gomisin G (max. 18.20 mg/100 g DM). The results from biomass extracts from *in vitro* cultures were compared with the results obtained from *S. henryi* leaf extracts. The lignan content of extracts from *in vitro* cultures was shown to be 13 times higher than that of parent plant leaf extracts [15].

**References**

[1] L. Havel, H. Vlašínová, I. Bohatcová, V. Trojan, J. Slanina, L. Březinová, Dibenzocyclooctadiene lignan production in *Schisandra chinensis* embryogenic culture, J Biotechnol. 136 (2008) S437. https://doi.org/10.1016/j.jbiotec.2008.07.1012.

[2] L. Březinová, H. Vlašínová, L. Havel, O. Humpa, J. Slanina, Validated method for bioactive lignans in *Schisandra chinensis in vitro* cultures using a solid phase extraction and a monolithic column application, Biomedical Chromatography. 24 (2010) 954–960. https://doi.org/10.1002/bmc.1391.

[3] H. Kohda, M. Ozaki, A. Namera, Production of lignans in calluses of *Schisandra* *chinensis*, J Nat Med. 66 (2012) 373–376. https://doi.org/10.1007/s11418-011-0586-y.

[4] A. Szopa, H. Ekiert, Lignans in *Schisandra chinensis in vitro* cultures, Pharmazie. 66 (2011) 633–634.

[5] A. Szopa, H. Ekiert, *Schisandra* *chinensis* (Turcz.) Baill. (Chinese magnolia vine) in vitro cultures., in: J.N. Govil (Ed.), Recent Progress in Medicinal Plants. Biotechnology and Genetic Engineering II, 39th ed., Studium Press LLC, USA, 2014: pp. 405–434.

[6] A. Szopa, H. Ekiert, Production of schisantherin A and gomisin G in in *vitro* cultures of *Schisandra chinensis*, Phytochem Lett. 11 (2015) 440–444. https://doi.org/10.1016/j.phytol.2014.12.022.

[7] A. Szopa, A. Kokotkiewicz, U. Marzec-Wróblewska, A. Bucinski, M. Luczkiewicz, H. Ekiert, Accumulation of dibenzocyclooctadiene lignans in agar cultures and in stationary and agitated liquid cultures of *Schisandra chinensis* (Turcz.) Baill., Appl Microbiol Biotechnol. 100 (2016) 3965–3977. https://doi.org/10.1007/s00253-015-7230-9.

[8] A. Szopa, H. Ekiert, The importance of applied light quality on the production of lignans and phenolic acids in *Schisandra chinensis* (Turcz.) Baill. cultures *in vitro*, Plant Cell, Tissue and Organ Culture (PCTOC). 127 (2016) 115–121. https://doi.org/10.1007/s11240-016-1034-1.

[9] A. Szopa, A. Kokotkiewicz, A. Król, M. Luczkiewicz, H. Ekiert, Improved production of dibenzocyclooctadiene lignans in the elicited microshoot cultures of *Schisandra chinensis* (Chinese magnolia vine), Appl Microbiol Biotechnol. 102 (2018) 945–959. https://doi.org/10.1007/s00253-017-8640-7.

[10] A. Szopa, A. Kokotkiewicz, M. Luczkiewicz, H. Ekiert, Schisandra lignans production regulated by different bioreactor type, J Biotechnol. 247 (2017) 11–17. https://doi.org/10.1016/j.jbiotec.2017.02.007.

[11] A. Szopa, A. Kokotkiewicz, M. Klimek-Szczykutowicz, M. Luczkiewicz, H. Ekiert, Different Types of In vitro Cultures of *Schisandra chinensis* and Its Cultivar (S. chinensis cv. Sadova): A Rich Potential Source of Specific Lignans and Phenolic Compounds, in: K. Ramawat, H. Ekiert, S. Goyal (Eds.), Plant Cell and Tissue Differentiation and Secondary Metabolites. Reference Series in Phytochemistry, Springer, Cham, 2020: pp. 1–28. https://doi.org/10.1007/978-3-030-11253-0_10-2.

[12] Y. Wang, G. Xi, Screening Culture Medium of *Schisandra sphenanthera* Rehd.et Wils’s Induction and Differentiation, Northern Horticulture. (2009).

[13] W. Liang, B. Deng, J. Xiao, S. Wang, Effects of basic media and culture conditions on callus growth and polysaccharide content in *Schisandra sphenanthera*, Journal of Central South University of Forestry & Technology. (2011).

[14] L. Wu, B. Xie, B. Deng, W. Liu, Y. Li, J. Lu, Callus Induction in *Schisandra sphenanthera* Rehd. Et Wils., Nonwood Forest Research. (2007).

[15] K. Jafernik, A. Szopa, M. Barnaś, M. Dziurka, H. Ekiert, *Schisandra henryi* C. B. Clarke in vitro cultures: a promising tool for the production of lignans and phenolic compounds, Plant Cell Tissue Organ Cult. 143 (2020) 45–60. https://doi.org/10.1007/s11240-020-01895-2.
